# Multi-dimensional investigation and distribution characteristics analysis of gut microbiota of different marine fish in Fujian Province of China

**DOI:** 10.3389/fmicb.2022.918191

**Published:** 2022-09-27

**Authors:** Hang Sun, Fangyi Chen, Hua Hao, Ke-Jian Wang

**Affiliations:** ^1^State Key Laboratory of Marine Environmental Science, College of Ocean and Earth Sciences, Xiamen University, Xiamen, China; ^2^State-Province Joint Engineering Laboratory of Marine Bioproducts and Technology, College of Ocean and Earth Sciences, Xiamen University, Xiamen, China; ^3^Fujian Innovation Research Institute for Marine Biological Antimicrobial Peptide Industrial Technology, College of Ocean and Earth Sciences, Xiamen University, Xiamen, China

**Keywords:** marine fish, gut microbiota distribution, diversity, host genetics, geographic factors

## Abstract

The gut microbiota plays an important role in animal health and behavior. In marine fish, the composition of the gut microbiota is affected by many complex factors, such as diet, species, and regional factors. Since more than one hundred fish species have been cultured in fish farms along with the 3,324 km coastline of Fujian Province in South China, we chose this region to study the gut microbiota composition of marine commercial fishes because sufficient different species, diets, and regional factors were observed. We investigated the distribution characteristics of the gut microbiota of seven cultured species (*Epinephelus akaara, Epinephelus coioides, Epinephelus lanceolatus* ♂ × *Epinephelus fuscoguttatus* ♀, *Siganus fuscescens, Pagrus major, Lateolabrax japonicus*, and *Acanthopagrus schlegelii*) living in the same aquatic region and one species (*E. akaara*) living separately in five regions separated by latitude. The impacts of diet, region, and species factors on fish gut microbiota were also evaluated. Diversity and multivariate analyses showed that the patterns of the microbiota were significantly different in different fish species within the same habitat and *E. akaara* with five latitude regions. Mantel analysis showed that AN, SiO_3_^2–^, DO, and NO_2_^–^ were the principal factors affecting the microbial community of *E. akaara* in the five habitats. Additionally, similar distribution characteristics occurred in different gut parts of different fishes, with an increasing trend of *Proteobacteria* and *Vibrionaceae* abundance and a decreasing trend of *Firmicutes* and *Bacillaceae* abundance from the foregut to the hindgut. *Vibrionaceae* was the most abundant family in the content. This study highlights that a persistent core microbiota was established in marine commercial fishes spanning multiple scales. The factors with the greatest effect on fish gut microbiota may be (i) host genetics and (ii) geographic factors rather than the microbiota in the diet and water environment. These core microbes regularly colonized from the foregut to the hindgut, which was driven by their underlying functions, and they were well adapted to the gut environment. Moreover, the microbiota in the content may have contributed more to the gut microbial communities than previously reported. This study could complement basic data on the composition of marine commercial fishes and facilitate relatively complete investigations, which would be beneficial for the healthy and sustainable development of aquaculture.

## Introduction

Gut microbiota have emerged as potent regulators of host metabolism and immunity (McElhanon et al., 2014; Sonnenburg and Bäckhed, 2016; Schoeler and Caesar, 2019). Most of the core microbial communities that colonize the gastrointestinal tract of vertebrates have different habitat characteristics (Zoetendal et al., 2012; Donaldson et al., 2016; Wen et al., 2019) and are closely related to many of their host genetics (Shabat et al., 2016; Kokou et al., 2019; Zeevi et al., 2019). However, the role of these communities and the interactions between important factors that constitute these communities, such as pH, temperature, diet, or host genetics, are not well understood. This process is also called environmental selection and genetic selection (Schmidt et al., 2019; Wen et al., 2019). In general, spatial transfer and maternal inheritance may further affect the composition of microbial communities and drive their distribution patterns (Ghoul and Mitri, 2016; Schulfer et al., 2018). The distribution characteristics of microbes are not only driven by the needs of microbiota but also inevitably affect the host health (Rehman et al., 2016; Hu et al., 2018), which has been observed for the microbiota in lower gastrointestinal tract of human, and they reflect the trends of dominant bacteria with differences in physiology and function (Donaldson et al., 2016).

As a classic aquatic species, fish account for more than half of all vertebrate species. They are not only an important aspect of biodiversity but also an important source of human–animal proteins (Lynch et al., 2016). Their gut microbiota would also affect the overall health of the host fish (Butt and Volkoff, 2019). However, compared with terrestrial organisms, fish are continuously exposed to various microorganisms in the environment. Thus, large variations have been observed between different studies based on the challenges of studying fish gut microbiota, which is mainly associated with the following three points: (1) influence of genetics and the environment, (2) sufficient availability of species for examination, and (3) need for better sample collecting methods and experimental methods (Baker et al., 2014; Gatesoupe et al., 2016; Yan et al., 2016; Egerton et al., 2018; Gang et al., 2018; Butt and Volkoff, 2019). Reports have indicated that there are gender differences in the gut microbiota of the *Gasterosteus aculeatus* and *Perca fluviatilis* (Bolnick et al., 2014). In addition, intraspecies differences in the composition of gut microbiota between individuals of the same species may exist in different habitats. For example, due to different habitats or diets, the bacterial groups in *Oncorhynchus mykiss* present significant differences (Navarrete et al., 2012). Moreover, despite being raised in the same environment, different bacterial populations were found in the tested freshwater larvae of *Hypophthalmichthys molitrix*, *Ctenopharyngodon idella*, *Aristichthys nobilis*, and *Megalobrama amblycephala* (Li et al., 2012). Compared with the stable methods that have been used in terrestrial animals, feasible methods of studying the fish gut microbiota are still being developed (Butt and Volkoff, 2019). Although considerable work has focused on understanding the roles of microbiome members in different gut locations in the past few years (Egerton et al., 2018; Gang et al., 2018; Kokou et al., 2019; Wen et al., 2019), their common distribution and habitat characteristics have not yet been understood. All studies are affected by different fish gut segmentation methods (McDonald et al., 2015; Gatesoupe et al., 2016; Yan et al., 2016; Egerton et al., 2018; Gang et al., 2018; Kokou et al., 2019; Le Doujet et al., 2019), which may affect the integrity of the fish microbiome. Choosing complete and sufficient fish gut fragments can fully reveal the distribution characteristics of gut microbes. Thus, correct experimental methods and sufficient samples are important for studying the gut microbiota in marine fish.

Inspired by the challenges of studying fish gut microbiota associated with the variety of biogeochemical processes in coastal water (Liu et al., 2017), we chose Fujian Province as a special investigation model because it has China’s second longest coastline and second largest fish mariculture. Many fish farms are located along with the coastal regions, and they raise more than one hundred commercial fish species. These farms are mainly distributed in six nearby cities [Zhangzhou, Xiamen, Quanzhou (QZ), Putian (PT), Fuzhou (FZ), and Ningde (ND)] (Ministry of Agriculture and Rural Affairs, 2019). According to statistics, the main commercial fish species farmed in Fujian Province are seabass, large yellow croaker, grouper, and sea bream, with an annual output of 4,788,249 tons (Ministry of Agriculture and Rural Affairs, 2019). Moreover, *E. akaara* is considered as one of the most popular and economically valuable grouper species, and it can be cultured in various locations in Fujian Province (Wang et al., 2016a). In this study, seven important fish species [*E. akaara* (SEa), *E. coioides* (Ec), *E. lanceolatus* ♂ × *E. fuscoguttatus* ♀ (Elf), *S. fuscescens* (Sf), *P. major* (Pm), *L. japonicus* (Lj), and *A. schlegelii* (As)] from the same area in Dongshan (DS) of Zhangzhou, one species [*Larimichthys crocea* (Lc)] from the ND coastal cultured areas, one species (*E. akaara*, Ea) from five habitats (along the coastline, from DS to ND, include DS, QZ, PT, FZ, and ND) and four gut fragments (foregut, midgut, hindgut, and content) were used as a model. We aimed to determine whether genetic or environmental factors play more important roles in shaping fish gut microbiota, identifying the distribution characteristics of the core microbial communities under multiple habitats, and elucidating the interaction between microbiota and gut fragments. These studies may complement the basic information and characteristics of the composition of gut microbiota in marine commercial fish and further help the aquaculture industry to improve fish health.

## Materials and methods

### Experimental design, sample sites, and sample collection

**FIGURE 1:**
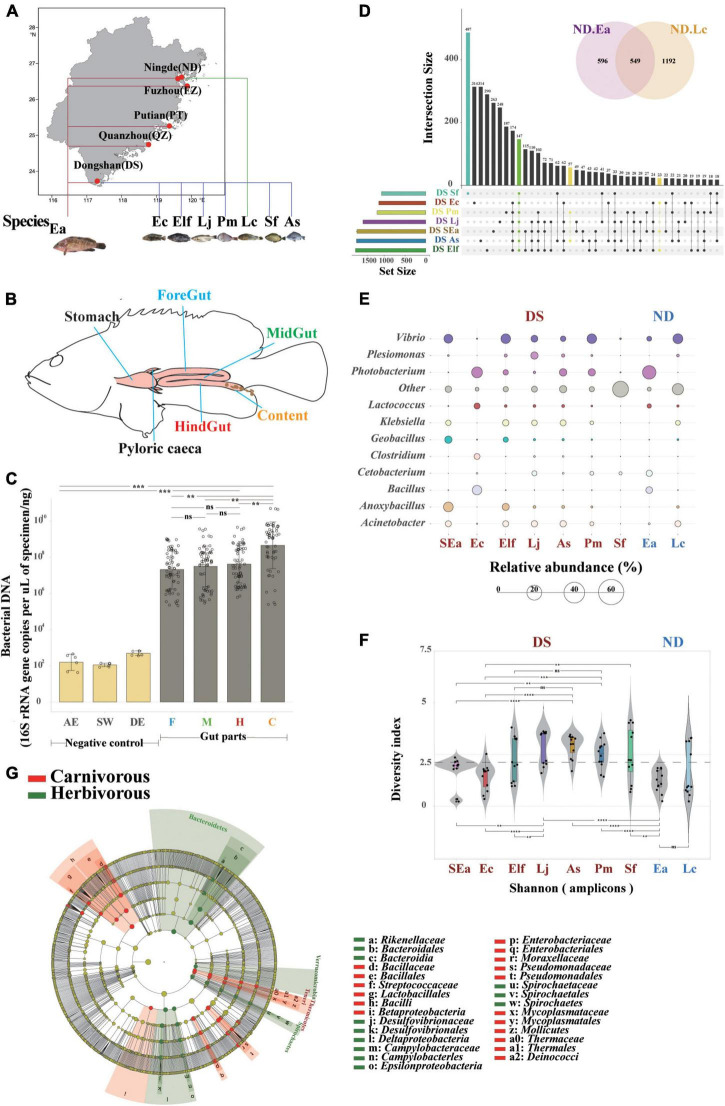
Host genetics play more important roles in the evaluation of core microbes of different commercial fish in the same area. **(A)** Study area and sampling sites in Fujian Province (from south to north: DS, QZ, PT, FZ, and ND). **(B)** Spatial structure of the digestive tract of the marine commercial fish (*E. akaara*) and our four sampling sites (foregut, midgut, hindgut, and content). **(C)** Bacterial loads (*n* = 68) were quantified by Q-PCR of the 16S ribosomal RNA gene. Differences were assessed by a two-tailed Student’s *t*-test. **p ≤ 0.05; ***p ≤ 0.005; ****p ≤ 0.0005; and *^ns^p* > 0.05. AE, AE-buffer used in DNA extraction; SW, sterile water used in tissue homogenization; DE, DNA extraction controls (empty bead tubes); F, foregut; M, midgut; H, hindgut; and C, content. **(D)** UpSet and venn diagrams showing shared or unique OTUs among different fish species, both in DS and ND. **(E)** Balloon plot showing the abundance of the core specificity communities across different fish species both in DS and ND at the genus level. **(F)** α-Diversity comparison based on the Shannon diversity index, with ANOVA used to determine significant differences (**p ≤ 0.05; ***p ≤ 0.005; ****p ≤ 0.0005; and *^ns^p* > 0.05). The plots are a combination of violin plots, dot plots, and box plots, with the box plots showing the median and the 25 and 75% quantiles. **(G)** Cladogram generated from linear discriminant analysis (LDA) effect size (LEfSe) showing the relationship between taxon (the levels represent, from the inner to outer rings, phylum, class, order, family, and genus).

A total of 108 fishes (9 in each group) and 72 water samples (0.5 m below the water surface and 9 parallel samples in each group) were collected in spring (from April 2018 to May 2018) from 6 sites along the coastline of Fujian Province in China. The detailed information and location for each site are shown in Figure 1A and Supplementary Table 2. Seven important agricultural fish species were obtained from DS city. SEa and Ec were collected from Tengsheng Breeding company that cultured in ponds with the same diet, while Elf, As, Pm, Lj, and Sf (Supplementary Table 1) were collected from a cage aquaculture company and were cultured in net cages with the same diet (some farmers would feed the same diet to reduce costs, and all diets were in line with the reality of aquaculture). Ea was collected from commercial hatcheries in four habitats (QZ, PT, FZ, and ND), and Lc was collected from the ND South China mariculture company and cultured in ponds. One liter of water was filtered for 16S rRNA sequencing through polycarbonate membranes with 0.2 mm pores (Millipore, MA, USA). Another 1 L water was used for physicochemical analysis. As the focus of our extensive analysis was the distribution of different microbial species and their potential relationships with physiological functions, we collected complete and sufficient gut parts to represent the microbial communities. For this purpose, we have redefined the gut segmentation methods to exclude the stomach and pyloric caeca (Figure 1B). The gut of each fish was dissected using sterile instruments and evenly divided into the foregut, midgut, and hindgut. The content of each section was gently squeezed out and collected together in a sterile cryotube. After dissection, each sample was ground, frozen, and stored at −80°C for further analysis. Each gut part of three parallel individual fish represented a research sample. The age of the fish was approximately 1 year (about 200 g in weight). At this stage, all fishes were sexually mature. During the entire trial, all treatments were performed similarly by the same trained research technician of the institute. After administering anesthesia using ethyl 3-aminobenzoate methanesulfonate salt analytical standard (0.1 g/L, 2 min immerse, MS-222, Sigma-Aldrich, USA), the fish guts were dissected using sterile instruments.

### Physicochemical factors analyses

Environmental variables, including pH, salinity, WT (water temperature), and DO (dissolved oxygen) were measured on site using Combo Water Quality Meter (86031, AZ Instrument Corp, China). Nutrient samples for NO_3_^–^, NO_2_^–^, AN (ammonia nitrogen) PO_4_^3–^, and NPOC (particulate organic carbon) detection after collection were immediately filtered through 47 mm GF/F filters and then frozen at −20°C. The samples for SiO_3_^2–^ detection were filtered through acid-cleaned 0.45-μm pore size acetate cellulose filters and stored at 4°C. Then, the samples for AN and SiO_3_^2–^ detection were stored in a 100-μL CCl_4_ solution. Nutrient samples were analyzed using a four-channel continuous-flow Technicon AA3 Auto-Analyzer (Bran-Lube GmbH, Germany) (Han et al., 2012). The copper–cadmium column reduction method was used to analyze NO_3_^–^ and NO_2_^–^ (Dai et al., 2008), while PO_4_^3–^ and SiO_3_^2–^ were measured using typical spectrophotometric methods. AN was run with a 722-type spectrophotometer (Xiamen Analytical Instrument Co., China) according to the indophenol blue spectrophotometric method (Pai et al., 2001). NPOC (*via* acid fuming) was determined using a PE-2400 SERIES IICHNS/O analyzer according to the JGOFS protocols (JGOFS, 1994). For chlorophyll measurement, 0.2–0.5 L water samples were filtered through 47-mm GF/F and extracted with 100% methanol at 4°C in darkness for 24 h. The extracts were centrifuged (5,000 g, 10 min), and the supernatant was scanned from 400 to 700 nm (DU800, Beckman, Fullerton, CA, USA). The chlorophyll concentrations were calculated using the previously described method (Porra, 2002).

### DNA extraction

The gut and content samples were separately thawed on ice and homogenized in liquid nitrogen, and ∼200 mg of each sample was used to extract the microbial genome. All DNA samples were extracted using QIAamp DNA Stool Mini Kit (Qiagen, Hilden, Germany) according to the manufacturer’s protocol (Eichmiller et al., 2016). In order to ensure the successful isolation of DNA, a Nanodrop 2000 UV-Vis spectrophotometer (Thermo Scientific, USA) was used to measure the concentration of DNA in the solution, and then the integrity of the DNA sample was evaluated by 1% agarose gel electrophoresis, and then the DNA was stored at −20°C for further analysis.

### 16S rRNA real-time Q-PCR

The content and gut parts DNAs were extracted as described. Q-PCR analysis was performed using SYBR Green master mix (Thermo Fisher, USA) and primers specific for the 16S rRNA sequence (forward, 5′-ACTCCTACGGGAGGCAGCAGT-3′, and reverse, 5′-ATTACCGCGGCTGCTGGC-3′) (Propheter et al., 2017). PCRs were quantified using standard curves generated from template controls of the specific primers. The statistical analysis was performed using a two-tailed Student’s *t*-test.

### 16S rRNA sequencing of the gut microbiome

Sequencing of the PCR-amplified V4 region (Kokou et al., 2019; Wen et al., 2019) of 16S rRNA was performed using a MiSeq 2000 Next Generation system (Illumina) at BGI Genomics Co., Ltd. (Shenzhen, China). Amplification of the V4 region using the primers 515F (5′-GTGCCAGCMGCCGCGGTAA-3′) and 806R (5′-GGACTACHVGGGTWTCTAAT-3′, each reverse primer contained a different 12 bp index) and the enzyme HotStar Taq (5 U μL^–1^; Qiagen), was performed under the following conditions: 94°C for 15 min, followed by 35 cycles of 94°C for 45 s, 50°C for 60 s, and 72°C for 90 s, as well as a final elongation step at 72°C for 10 min. The PCR products of each sample were detected by electrophoresis on 2% agarose gel and the 380 bp bright main strip was purified by using QIAquick Gel Extraction Kit (QIAGEN, Germany). Then, the concentrations of purified PCR products were measured by NanoPhotometer^®^ Classic Launched (IMPLEN, Germany). After purification and quantification, the amplicons from each reaction mixture were mixed in equal amounts based on the concentration, and then the amplicon libraries were generated.

### Sequences data processing

The Quantitative Insights Into Microbial Ecology (v1.8.0) pipeline (Caporaso et al., 2010) was used for data quality control (QC) and analysis. In brief, low-quality reads that met the following criteria were discarded: reads with an average PHRED score of less than 20, read lengths shorter than 150 bp, sequence containing unknown bases or primer mismatches, and mononucleotide repeats exceeding 8 bp. High-quality data were obtained and overlapped with tags using FLASH software. The USEARCH (v9.0) (Edgar et al., 2011) software was used to cluster the tags into operable taxonomic units (OTUs) using a 97% identity threshold. Representative sequences of OTUs were obtained and compared using the Greengene database (v.13.8) (McDonald et al., 2012) and RDP Classifier (v2.2) software for taxonomic annotations. The confidence threshold was set to 0.5. Due to the variation in sequence depths among samples, all samples were normalized to the lowest depth by subsampling (6,000 reads per sample). The OTU abundance of each sample and the six-level taxonomic classification from phylum to species were then obtained.

### Comparison of gut communities and bioinformatics analysis

The qualified OTU data were used to calculate α-diversity metrics of the Shannon index, Chao1, and Observed species (richness) by using QIIME software package (Caporaso et al., 2010), which were determined by ANOVA with Bonferroni’s post hoc test using SPSS software (SPSS, Chicago, IL, USA). Bray–Curtis dissimilarities were produced as β-diversity measures and then subjected to principal coordinate analysis (PCoA) with the vegan package and QIIME software package (Caporaso et al., 2010). The different gut parts were statistically compared through the similarity analysis (ANOSIM). The overlap of the microbial communities was determined by the *R* values from the ANOSIM, according to the method from Buttigieg and Ramette (2014), with *R* > 0.75 indicating well separated, 0.50 < *R* ≤ 0.75 indicating separated but overlapped, 0.25 < *R* ≤ 0.50 indicating separated but strongly overlapped, and 0.25 = *R* indicating barely separated. Principal components analysis (PCA) was employed to evaluate the differences in microbial community composition between herbivorous and carnivorous fish using Bray–Curtis distances with the vegan packages (Dixon, 2003). The similarity analysis (PERMANOVA) was performed to examine the differences among the microbial community composition (Clarke and Warwick, 1994). The divergence of microbial communities between carnivorous and herbivorous fishes was determined by linear discriminant analysis (LDA) effect size (LEfSe), which is a biomarker discovery and explanation tool for high-dimensional data (Segata et al., 2011). Microbiota-based biomarker discoveries were done with LEfSe using the online galaxy server.^1^ The LDA scores derived from LEfSe analysis (Segata et al., 2011) were used to show the relationship between taxon using a cladogram (circular hierarchical tree) of significantly increased or decreased bacterial taxa in the gut microbiota between carnivorous and herbivorous fishes. Levels of the cladogram represent phylum, class, order, family, and genus from the inner to outer rings. The color codes indicate the groups, and the letters indicate the taxa that contribute to the uniqueness of the corresponding groups at an LDA of >2.0.

The co-occurrence network of microbial communities in Ea with different habitats was constructed. In order to visualize the associations in the network, we constructed a correlation matrix by calculating the possible pairwise Spearman’s rank correlations. A valid co-occurrence was considered to have a statistically robust correlation between species with Spearman’s correlation coefficient (*r*) > 0.6 and the *p*-value < 0.01 (Jiang et al., 2017a). The nodes in the reconstructed network represented bacterial taxa (OTUs), and the edges represented the high and significant correlations between the nodes. To describe the complex pattern of interrelationships of bacterial OTUs, the topological characteristics of the networks were calculated as follows: average path length (APL), graph density, network diameter, average clustering coefficient (avgCC), average degree (avgK), and modularity (M). Network analyses were performed using the dplyr, vegan, and psych packages in R software (Jiang et al., 2017a). The correlation networks were visualized using Gephi software.

Mantel test was performed to detect the correlation between environmental variables and microbial communities in different regions using the ggcor R package. Canonical correspondence analysis (CCA) was also conducted to determine the impact of environmental factors on the microbial composition of fish gut in the same region through vegan and ggplot2 packages. The microbial source tracking (FEAST) (Shenhav et al., 2019) was applied to predict the sources of the microbial communities detected in each of the five locations (foregut, midgut, hindgut, content, and water), based on the code and version at https://github.com/cozygene/FEAST/tree/FEAST_beta. Prediction of the kyoto encyclopedia of genes and genomes (KEGG) pathways in samples was performed with PICRUSt software (McDonald et al., 2015). Statistical analysis of metagenomic profiles (STAMP) were used to recognize the abundance differences of KEGG pathways among different gut parts (Parks et al., 2014). Other bioinformatics analyses based on the predicted functions of the KEGG pathways were consistent with the methods for OTUs.

All analyses were conducted in R (version 3.5.1, R Development Core Team) unless otherwise stated.

## Results

### Host genetics play more important roles in the evaluation of core microbes of different commercial fish in the same area

To identify the distribution characteristics and influence of genetic and environmental factors on fish gut microbiota, we first defined four gut parts (foregut, midgut, hindgut, and content) that can fully reflect the gut microbiome of 108 fish individuals (Figures 1A,B, Supplementary Figure 1, and Supplementary Table 2, the research design is described in section “Material and methods”). To confirm the applicability of intestinal sampling methods in this study, we measured the total 16S rRNA gene copy numbers by absolute quantitative PCR to compare the bacterial loads in the gut segments of all fishes. The bacterial load trends of the foregut, midgut, and hindgut were the same (*p* > 0.05), while the number of bacteria in the content increased significantly (Figure 1C). We also compared the bacterial DNA composition of the gut tissues with negative control specimens, which showed that the extraction method did not have an effect (Dickson et al., 2018). These findings further strengthen the view that the microbial communities colonized equally along with the different gut parts, which suggested that one or two gut parts might not be complete and fully represent the characteristics and community composition of the fish gut microbiome. Here, we obtained a total of 12,761,035 quality-filtered sequences from 108 fishes, with an average of 78,182 reads (Supplementary Tables 3, 4), and 5,691 OTUs (operational taxonomic units) were then clustered based on 97% sequence identity.

Then, we conducted a comparative experiment in which four gut parts (both foregut, midgut, hindgut, and content) were sampled together to analyze their core microbes and regarded them as representative of the entire fish gut microbiome. To avoid the influence of environmental factors, we defined the influence of genetic factors on gut microbiome with similar ecological niches of different fish species, such as SEa, Ec, Elf, Sf, Pm, Lj, and As in DS, Ea, and Lc in ND. An OTU set analysis of the gut microbial communities of each fish showed differences (shared/total: 147/1,741, Figure 1D), and Sf had the highest specificity. Interestingly, the fishes in the same genus did not show the same features beyond the abundance analysis, which may indicate the complex composition of gut microbes in individual fish species at the species level. Then, we implemented a previously described method for detecting stable residents of the microbiome (Schoeler and Caesar, 2019) to examine these core microbes (relative abundance above 5%) of farmed fishes, and both family and genus level annotations could be specifically found in different fish species (Figure 1E, Supplementary Figures 2, 3). We also found that certain specific genera of microbes only occurred in one fish species (Supplementary Figure 4), although presented a relatively low relative abundance (approximately 0.3%). The divergence of these fish species in the same habitats suggests that the microbiota is closely related to host genetics, making them ideal candidates for studying differences in the core microbiome between different species. In addition, when we examined the divergence between different fish families (Figure 1E), we found that the diversity of Lateolabracidae and Sparidae was significantly higher than that of Epinephelinae and Sciaenidae (Figure 1F). The divergence of these fish species in the same habitats suggest that they are closely related to host genetics.

*Siganus fuscescens* is one of the most important components of marine commercial herbivorous fishes (Michael et al., 2013; Nielsen et al., 2017) in Fujian Province. The differences in the microbial composition between carnivorous and herbivorous fishes with the same diet are an interesting topic because the diet is one of the strongest influencing factors on gut microbiota (Shaani et al., 2018). The diversity between carnivorous and herbivorous fishes was significantly different (Supplementary Figure 5). The results of the LEfSe analysis support these results and indicated that the relative abundance of *Rikenellaceae*, *Desulfovibrionaceae*, *Campylobacteraceae*, and *Spirochaetaceae* was higher in herbivorous fishes while the abundance of *Bacillaceae*, *Streptococcaceae*, *Enterobacteriaceae*, *Mycoplasmataceae*, and *Thermaceae* was higher in carnivorous fishes (Figure 1G). We next examined the core microbes in each fish species at the genus level (relative abundance above 5%) to identify the preference for host genetics between carnivorous and herbivorous fishes (Supplementary Figure 6). *Desulfovibrio*, *Arcobacter*, and other were significantly more abundant in herbivorous fishes, while *Vibrio*, *Photobacterium*, *Enterovibrio*, *Acinetobacter*, *Klebsiella*, *Lactococcus*, *Bacillus*, *Oceanobacillus*, *Clostridium*, *Anoxybacillus*, *Geobacillus*, *Pseudomonas*, *Plesiomonas*, *Cetobacterium*, *Fusobacterium*, and *Shewanella* were relatively more abundant in carnivorous fishes. Notably, unclassified bacteria (other in Figure 1E) of Sf at the genus level reflected the complex features and technical limitations of identifying the microbial communities in herbivorous fishes. These findings further strengthen the idea that host selection may play more important roles in enabling the establishment of microbiota colonization in the guts of fish with similar cultured environments and the same diet.

### Gut microbial communities of the same marine commercial fish (*Epinephelus akaara*) will also be affected by different habitat factors

**FIGURE 2:**
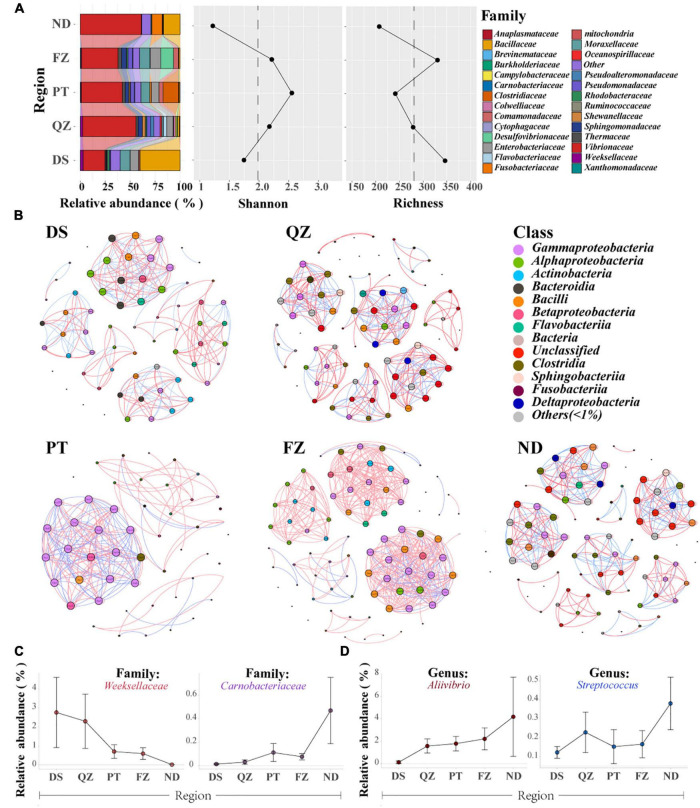
Gut microbial communities of the same marine commercial fish (*E. akaara*) will also be affected by the factors of different habitats. **(A)** Divergence of microbial communities among the five habitat groups (DS, QZ, PT, FZ, and ND) of Ea, with the relative abundance, richness, and Shannon index analysis. Only the dominant microbial family with top 28 of each group are plotted. **(B)** Networks of co-occurring bacterial OTUs in Ea from the five habitat groups based on the correlation analysis. The co-occurring networks are colored by class. A red edge indicates a positive interaction between two individual nodes, while a blue edge indicates a negative interaction. **(C)** Relative abundance of the specificity patterns from south to north at the family level. **(D)** Relative abundance of the specificity patterns from south to north at the genus level.

Different habitats of the marine environment for wild fishes may contribute to the establishment of unique microbial community characteristics, such as distinct life cycles, migration patterns, and feeding resources (Xia et al., 2014; Jones et al., 2018). These characteristics are especially true for the diversity of the same fish in different ecotypes. Inspired by this, we examined whether Ea farmed in five habitats (cultured in ponds from south to north in Fujian Province) have the same patterns as wild fish without genetic factors. We found that the core microbial communities are distinct in Ea from different habitats at the family level; furthermore, the species richness and diversity also supported this difference (Figure 2A). Then, based on the strong and significant correlations, we explored the bacterial co-occurrence patterns among Ea from the five habitats using network analysis (Jiang et al., 2017a,b). Overall, the ecological networks were markedly different among the different habitats. In these five networks, the number of positive correlations was much higher than that of the negative correlations (number of positive correlations >65%, Figure 2B and Supplementary Table 5). Significant differences were observed among the graph density, avgCC, avgK, average weighted degree, and modularity in these empirical networks. These results suggested that the composition of the bacterial communities was obviously different among each habitat, and the empirical networks had prominent “small-world” modularity and hierarchy of their topological properties. Further structural analysis showed that a deterministic pattern of intrafamily co-occurrence was prevalent in the bacterial networks. The bacterial OTUs in the dominant families, such as *Gammaproteobacteria*, *Alphaproteobacteria*, *Actinobacteria*, *Bacilli*, and *Bacteroidia*, tended to co-occur more frequency than that of other families.

Notably, we found some interesting patterns both at the family and genus levels along with the coastline (Figures 2C,D). *Weeksellaceae* and *Carnobacteriaceae* were detected in almost all fish samples, and they showed a downward trend and upward trend from south to north of Fujian Province along with the coastline, respectively. At the genus level, both *Aliivibrio* and *Streptococcus* showed an upward trend from south to north. Although these fishes belong to the same species and were farmed, these findings further suggested that the core microbes of cultured Ea are distributed in niches, thereby adapting to the regional influences.

### Habitat filtering by genetic selection and water environmental factors may be the major driver shaping the gut core microbial communities of marine commercial fish with similar diets

**FIGURE 3:**
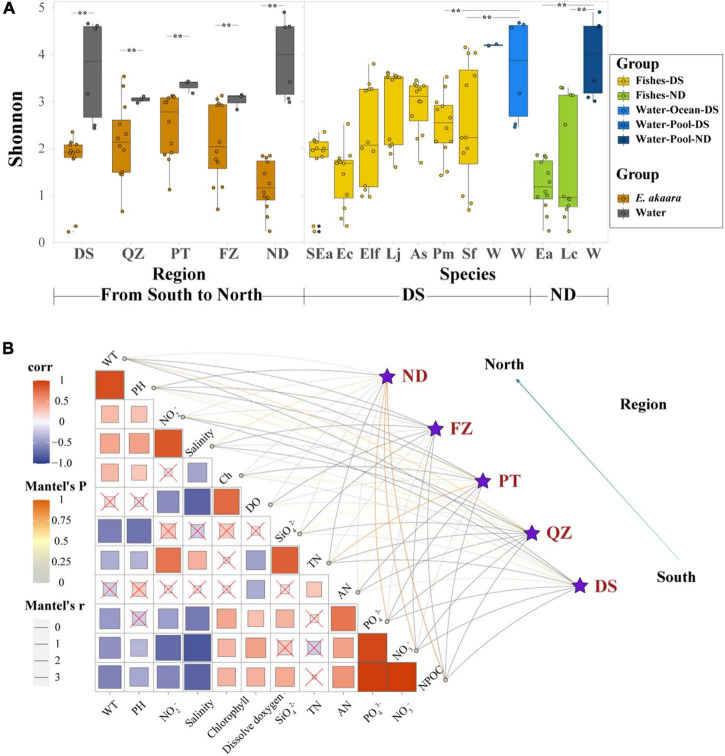
Habitat filtering by genetic selection and water environmental factors may be the major driver shaping the gut core microbial communities of marine commercial fish with similar diets. **(A)** α-Diversity comparison based on the Shannon diversity index with dot plots and box plots. Each fish group represents 9 fish individuals mixed by 12 sample individuals, each water group represents 3–6 samples depending on their aquaculture environment, **p < 0.05. **(B)** Environmental drivers of the microbial communities from south to north (Bray–Curtis distance) and environmental factors were analyzed with Mantel tests. The edge width corresponds to the *R*-value, and the edge color denotes the statistical significance. The color gradient indicates Pearson correlation coefficients among the environmental factors indicate no significant correlation at 0.05 level.

According to the significant differences in the microbial composition of the same fish in Fujian Province, we expected to find important factors affecting the gut microbial communities. Due to the associated well-defined physiological thresholds, environmental factors and dietary composition limit the range of activities of most animals (Bates et al., 2013). Inspired by this, we examined the core microbial communities of the water environment and forage and the interaction between environmental factors and gut microbial communities. All the α-diversity indices of the microbial communities in water were significantly higher than that of the diversity in the fish gut (Shannon index, *p* < 0.05; Figure 3A). However, the core microbiota of the fish gut, forage, and water environment could not simply reflect the interaction with high divergence in the abundance of core microbiota (Supplementary Figures 7, 8).

We next examined whether different environmental factors of aquaculture water affect the microbial communities, which would indicate the preference of microbes for specific habitats. The DS microbial community exhibited a significant correlation with pH, salinity, SiO_4_^2–^, and AN (*p* < 0.01) (Figure 3B). The QZ group exhibited a significant correlation with WT, chlorophyll, and DO (*p* < 0.01), while PT group exhibited a significant correlation with NO3^–^, DO, SiO_4_^2–^, and TN (*p* < 0.01). FZ group showed a significant correlation with WT, TN, and AN (*p* < 0.01). It is worth noting that all the correlations between Ea and different habitats were positive, which indicated the characteristics of the water environment for aquaculture. While the CCA ordination showed that PO_4_^3–^, NO_3_^–^, and NO_2_^–^ were the main interaction factors of different fish species in similar ecotypes (Supplementary Figure 9), PO_4_^3–^ and NO_3_^–^ showed a stronger correlation in Epinephelinae. These finding further indicated that water environmental factors might be important driving forces in shaping the microbial communities of marine commercial fish guts when the fish are fed similar diets.

### Common distribution characteristics of microbial communities in different gut fragments

**FIGURE 4:**
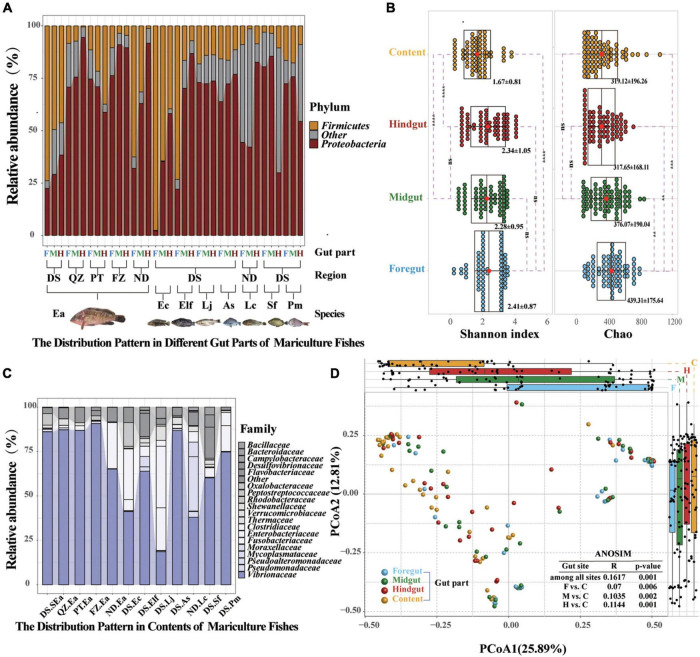
Common distribution characteristics by gut parts are visible shaping marine commercial fish microbial communities. **(A)** Relative abundance of the microbial communities at the phylum level found in different gut compartments (foregut, midgut, and hindgut). Each group represents nine fish individuals with a parallel sample mixed by three individuals. Only *Proteobacteria* and *Firmicutes* of different gut parts are plotted. **(B)** Relative abundance of the microbial communities at the family level found in the content. Only the dominant microbial family with top 20 of the content are plotted. **(C)** α-Diversity comparison based on the Shannon diversity index in each of the parts using ANOVA to determine significant differences (**p ≤ 0.05; ***p ≤ 0.005; ****p ≤ 0.0005; and *^ns^p* > 0.05). In data shown as a combination of dot plots and box plots (*n* = 108 fish individuals), with the center red point indicates the mean value in the corresponding group, and the data are expressed as the means ± SD. **(D)** Principal coordinate analysis plot generated using OTU metrics based on the Bray–Curtis dissimilarities. Each point represents a sample. Differences were assessed by ANOSIM and significance was established at *p* < 0.05. An *R*-value close to “1” suggests dissimilarity between groups, whereas an *R*-value close to “0” suggests an even distribution of high and low ranks within and between groups.

After the multidimensional investigation, we sought to identify the distribution characteristics of gut microbiota in fish individual. If each part provides different functional requirements and conditions and diet, environment, and habitat filtering functions are established, then the distribution patterns and characteristics of each part should be revealed. We compared these four selective forces and their relative effects on the microbiome composition of different gut parts (Turnbaugh et al., 2009; Baker et al., 2014; Fetissov, 2017; Egerton et al., 2018; Gang et al., 2018). Amplicon sequencing showed that the distribution patterns in different gut parts were significantly different, as indicated by the increasing and decreasing trends of *Proteobacteria* and *Firmicutes* from the foregut to the hindgut, respectively, and similar increasing and decreasing trends of *Vibrionaceae* and *Bacillaceae*, which have been confirmed in most fish species (Figure 4A and Supplementary Figure 10). Moreover, we found that *Proteobacteria* (59.2–92.28%) and *Vibrionaceae* (37.99–90.7%) were the most abundant strains in terms of content (Figure 4C and Supplementary Figure 11). Among the four gut parts, the content showed significantly lower diversity and the foregut showed the highest richness (Figure 4B). A PCoA was then conducted to visualize the differences in taxon composition among different sites, and the difference between the midgut and hindgut group was also greater than that of the foregut group (Figure 4D). These findings further confirm that the microbial communities presented characteristic colonization features along the different gut parts of marine commercial fish.

### Microbiome of the content may originate in the midgut and hindgut

**FIGURE 5:**
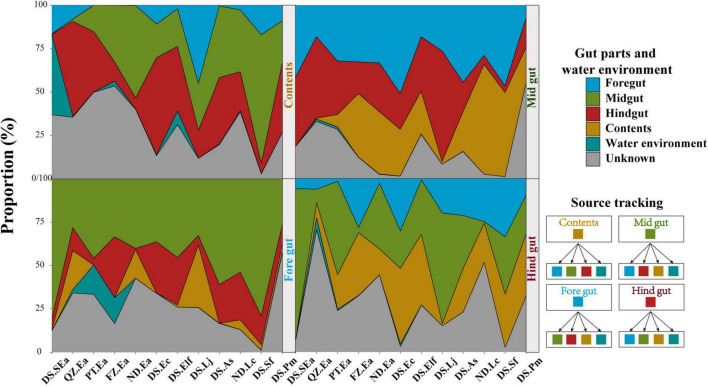
Tracing the source of gut parts in each gut part and water environment. Each gut part and water environment were taken as potential sources of other gut parts. The bands of each color indicate each group.

Interestingly, the results in Figure 4 indicated that the distribution characteristics of bacterial taxa in different gut parts might highlight the role of gut microbiota. Reports have indicated that the diazotrophic population colonized in the hindgut of Amazonian catfish may play important roles in nitrogen fixation (McDonald et al., 2015), thus indicating the value of studying whether microbes colonize different gut parts to perform their specific functions. To track the potential origin of the microbiome in the gut parts, we examined the possible sources, including the four gut parts and water environment (Figure 5). We found that the microbes of the content mainly originated in the midgut and hindgut, while the microbes in the foregut mainly originated in the midgut. However, the potential source of the water environment only fluctuated slightly in the content and foregut and the proportion was low.

Then, bacterial KEGG pathways were used to preliminarily predict whether the potential functions of the microbes in the four gut parts were different (Langille et al., 2013). Cellular processes and environmental information processing-related functions, such as cell motility, the abundance was significantly higher in the content, indicating the higher motility of microbes in the content (Supplementary Figures 12a,b). The functional genes related to diseases increased significantly in the foregut, while the proportion of functional genes related to metabolism in the midgut and hindgut was higher (Supplementary Figures 12c–f). These findings suggested that the normal colonization of the microbial communities might be driven by the potential functions of different gut parts.

## Discussion

In many vertebrates, changes in the gut microbiota have important impacts on host health (Bäckhed et al., 2005; Mayer et al., 2015; Sharon et al., 2016). Thus, it is meaningful to compare the gut microbiota compositions of diseased and healthy groups or beneficial additive groups and then correlate the findings with clinical markers (Turnbaugh et al., 2009). However, even in humans, diet, genetic, and environmental factors have always been important determinants in driving intestinal community composition (Fallani et al., 2011; David et al., 2014; McElhanon et al., 2014; Gomez et al., 2016; Rinninella et al., 2019). In fish, due to the complexity of marine habitats, more issues need to be considered to illustrate the factors that shape the gut microbiota (Egerton et al., 2018). Because of the rich biological resources in Fujian Province, which has a coastline of 3,324 km, different survival rates and meat yields will occur among different fish species cultured in the same region and the same fish cultured in different regions (Fujian Provincial Department of Ocean and Fisheries, 2013). Inspired by the studies on the different genetics of different fish species, different nutritional and metabolic characteristics, and different environmental factors of different culture regions, experimental methods and locations were designed to further understand the composition of fish gut microbiota and provide basic information on marine aquaculture in China.

Since the environment and diet composition are considered the main factors affecting the gut microbial communities (Donaldson et al., 2016; Egerton et al., 2018; Shaani et al., 2018), we first established a comparative model with similar habitat and diet characteristics to evaluate the diversity of the core microbes of eight marine commercial fish. Although these fish have different genetic factors, life habits, and metabolic characteristics, we analyzed the impact of genetic factors on gut microbiota based on fish that lived in the same cultured region and were fed the same diet. At the genus and family levels, the abundance of core microbes in different fish species was significantly different. These differences could also be found in different fish families. Moreover, fish living in net cages showed more unique gut microbial genera (Supplementary Figure 4) compared with fish living in cement pools, which may indicate that the complexity of gut microbiota diversity may be achieved through adapting and buffering the environment to help the host survive (Butt and Volkoff, 2019). Interestingly, despite receiving the same diet, a highly complex and variable gut microbial community was observed in herbivores (Figure 1G). The proportion of unclassified results at the genus level was 70.87%, which also indicated that the composition was more complex (Pitt, 1997; Nielsen et al., 2017; Jones et al., 2018). These findings further demonstrated that the core microbes may be widely affected by the host genetics of different fish species with similar diets in the same region, such as Atlantic salmon (*Salmo salar Linnaeus*), European seabass (*Dicentrarchus labrax*), tilapia (*Oreochromis mossambicus*), and perch (*Perca fluviatilis Linnaeus*) (Günther et al., 2016; Kokou et al., 2019).

After investigating different fish species in the same niche, we sought to determine whether the divergence occurred in different habitats of the same species. However, compared to wild populations, little is known about the microbiota composition of the same fish living in different farmed habitats (Jones et al., 2018; Le Doujet et al., 2019). Although these fish (Ea) were cultured in different regions with different environmental factors, they have the same genetic factors and similar dietary structure and metabolic characteristics. We then investigated the microbial communities of Ea living along the coastline of five cities (DS, QZ, PT, FZ, and ND) with similar aquaculture environments. The core microbes of the cultured Ea showed great differences and were also distributed based on their ecological niches to adapt to the regional influences, which are similar to the geographic factors that affect the species-specific microbial communities of wild fish (Kellogg et al., 2017; Jones et al., 2018; Shaani et al., 2018; Le Doujet et al., 2019). A network analysis was performed to provide a comprehensive understanding of the interactions, compositions, and assembly roles in the fish microbial community that reflect the ecological processes in the fish microbial community (Layeghifard et al., 2017). The networks of Ea from different habitats were significantly different and strengthened the divergence of Ea cultured in different habitats. We found that the proportion of positive interactions in the fish gut of all habitats was high and thus beneficial to improving the stability of the interaction networks and helping the microbial community adapt to environmental changes (Krause et al., 2003; Le Doujet et al., 2019). These strong interactions between intra- and interphylum taxa were found in all habitats of Ea (Supplementary Table 5), thus providing new evidence for the influence of geographic and taxonomic factors on the assembly of microbial community in cultured fish. It is worth noting that we also found some microbes with regional correlation patterns from south to north (Figures 2C,D), because they were affected by environmental factors, such as the pattern of distance-decay (Tuomisto et al., 2003; Martiny et al., 2011). Therefore, regional factors may explain the different microbiota composition and interspecies interaction models in the same fish farmed along with the coastline. It is worth noting that when we analyzed the core microbes by gathering the four gut parts as representative of the fish gut microbiota, the results were obviously different from the composition of fish gut microbes in other studies (Li et al., 2012; Navarrete et al., 2012; Baker et al., 2014; Gatesoupe et al., 2016; Egerton et al., 2018; Gang et al., 2018; Le Doujet et al., 2019). The divergence of these core microbes complemented the information and characteristics of the composition of gut microbiota in marine commercial fish.

Due to various regional factors (such as geography, culture, microbial diversity, and physicochemical factors), it is important to evaluate the microbial diversity and physicochemical factors in water when studying the microbial composition of marine commercial fish (Jeraldo et al., 2012; Lek et al., 2012; Bates et al., 2013; Egerton et al., 2018). Here, we aimed to reveal the factors that influence and shape the microbial communities in marine commercial fish. Although the diversity of water microbiota was significantly higher than that of fish, there was always a clear difference between the microbial composition of the fish gut and water environment (Figure 3A). These findings indicated that the interaction of microbiota between the fish gut and water environment may be greatly limited. Therefore, the microbiota of the fish gut could not simply reflect the environmental microbiota, and the abundance of the shared OTUs was very low (Bakke et al., 2013; Baker et al., 2014). Simultaneously, the interaction of microbiota between the fish gut and diet may also be limited (Supplementary Figure 7), although the microbial communities can be changed based on the diet composition (Chen et al., 2014; López Nadal et al., 2020). Source tracker data showed that only part of the microbiota from the contents, and foregut was derived from the water environment, although the proportion was low (4.97% of content and 2.97% of foregut on average) (Figure 5). In addition, we found that these 12 environmental factors were significantly correlated with the microbial communities of Ea from south to north, such as NPOC, PO_4_^3–^, and NO_3_^–^ in the ND group. These changes in microbial communities could be explained as follows. First, NO_3_^–^, PO_4_^3–^, NO_2_^–^, and SiO_3_^2–^ are important factors in primary production and also effective nutrients for stimulating bacterial growth (Jiao et al., 2017). Suspended solids (NPOC) and chlorophyll could act as a source of nutrients for aquatic microorganisms and provide more habitats for the growth of bacteria (Na et al., 2014; Zhou et al., 2020). WT, DO, AN, and pH have always been considered to be the key environmental variables that shape the microbial community structure in the aquaculture environment (Zhao et al., 2018). These findings further indicated that physicochemical factors may play more important roles in shaping fish microbial communities compared with the microbial diversity of water environment.

The microbiome in internal organs is usually linked with the specific roles of the organs (Donaldson et al., 2016; Marchesi et al., 2016; Kokou et al., 2019; Wen et al., 2019). For example, *Bacteroides* spp. in the human colon can be used to absorb and consume fatty acids and simple carbohydrates in food (Koropatkin et al., 2012; Donaldson et al., 2016). However, the studies of the fish gut microbiome have adopted a variety of gut segmentation methods and experimental methods, which may provide a limited understanding of fish microbial communities and their potential functions (Baker et al., 2014; Gatesoupe et al., 2016; Yan et al., 2016; Egerton et al., 2018; Gang et al., 2018; Kokou et al., 2019). In the present study, the four gut parts could be regarded as representative of the fish gut, and almost all segmentation methods were covered. We evaluated the differences in the microbiota of the foregut, midgut, hindgut, and contents of eight fish species. Here, significant distribution patterns of core microbes were found from the foregut to the hindgut, which was ignored in earlier studies. These findings further suggested that the distribution of the fish gut microbiota has a certain trend, just like the distribution in other terrestrial vertebrates (Wen et al., 2019). In addition, we observed that the microbiota in content was very different from that of the other segments, among which *Proteobacteria* and *Vibrionaceae* were the main microbiota in all fishes investigated in this study. This finding is consistent with other studies on the microbiota of fish, such as *Gadus morhua*, *Poecilia reticulata*, and *Scophthalmus maximus* (Sullam et al., 2012; Xing et al., 2013; Le Doujet et al., 2019), but is different from other vertebrate species, such as the high abundance of *Firmicutes* and *Bacteroidetes* in the fecal community of humans and chickens (Donaldson et al., 2016; Wen et al., 2019). Most of the microbiome present in the contents are considered to be transient, allochthonous, and free-living bacteria, which usually do not come into contact with the mucosal surface of the gut (Egerton et al., 2018; Le Doujet et al., 2019). However, we found that the abundance of most microbes in the hindgut affected the bacteria in the contents (Figure 4). The results of source tracking (Shenhav et al., 2019) also showed that the microbes of the contents mainly originated in the midgut and hindgut (Figure 5). These findings further suggested that the microbiota of the contents came from the gut of fish and were not just allochthonous bacteria. Furthermore, the bacterial loads in the foregut, midgut, and hindgut did not show a different pattern (Figure 1C). These results further confirmed the idea that these four gut parts are not only necessary for studying the spatial distribution of microbes but also essential for analyzing the diversity of the gut microbiota of the entire fish species. From the perspective of digestion, host nutrition, and immunity, both the contents and gut mucosa-associated bacteria have become important members of the microbial community (Smith et al., 2015).

As mentioned above, the microbial community is regularly distributed and highly adapted to the environment of a specific gut location. However, these distribution characteristics and selectivities have not been fully understood in previous studies focused on host genetics, functions, or environmental factors in the gut (McDonald et al., 2015; Wang et al., 2016b; Egerton et al., 2018; Le Roy et al., 2018). The intestinal microbiome plays an important role in facilitating digestion and metabolism, intestinal development, and resistance to pathogens (Pérez et al., 2010; Flint et al., 2012; Egerton et al., 2018). Therefore, we predicted the functions of the core microbiota in the four different gut parts. It should be noted that the potential functions of microorganisms predicted here may represent an auxiliary tool for understanding the role of the fish gut microbiota (Zhao et al., 2018). Our results showed that due to the relatively high abundance of potential pathogens, such as *Vibrionaceae* (15.1–87.9%), the metabolism-related functions of the microbiota in these fishes were significantly increased in the hindgut. In the foregut, there were significantly more disease-related functional microbiota, which may be due to the relatively higher abundance of potential probiotics, such as *Bacillaceae* (3.35–69.6%). Numerous studies have confirmed that when pathogens cause inflammation by altering the intestinal environment, the colonization of probiotics has a positive effect on immune regulation (Pérez et al., 2010; Tlaskalová-Hogenová et al., 2011; Otles, 2014; Bäumler and Sperandio, 2016; Egerton et al., 2018). Potential functions associated with cell motility were higher abundance in the contents, which might be due to the high motility of free-living bacteria and their important roles in metabolism and digestion (Supplementary Figures 12a–f). It is worth noting that the midgut microbiota is an important link between other gut parts. The results highlighted that the selective function was an important determinant of core-microbe colonization, which would potentially contribute to their survival and functions.

## Conclusion

In summary, our results systematically complement previous insights into the composition of core microbes in marine commercial fish gut based on a multidimensional investigation. The great impact of host genetics and environmental factors on the specificity of core microbes in fish gut has been proven by restricting conditions, and they have a more important impact than the microorganisms in the culture environment and diet. Moreover, these core microbes regularly colonized from the foregut to the hindgut driven by their underlying functions. The content is also an important part in the study of the fish gut microbiota. Due to the complexity of the aquaculture environment, diet composition, and fish species, it is necessary to study the fish gut microbiota by multidimensional investigations, and the findings will be beneficial for the healthy and sustainable development of aquaculture. In addition, an investigation of the time scales (different seasons) and the dynamic variability of nutritional factors will be reported in our future studies.

## Data availability statement

The data presented in the study are deposited in the NCBI repository, accession number PRJNA718646.

## Ethics statement

The animal study was reviewed and approved by the Xiamen University.

## Author contributions

K-JW and HS conceived and designed as well as analyzed the experiments. HS performed all the experiments and wrote the manuscript. FC assisted with the writing and contributed to the preparation of the figures. HS and HH provided the investigation and sample collection. K-JW contributed all of reagents, materials, and analysis tools and wrote the manuscript. HH arranged several experiments of mariculture. All authors reviewed the results and approved the final version of the manuscript.

## Footnotes

1
https://huttenhower.sph.harvard.edu/galaxy/

## References

[B1] Bäckhed F. Ley R. E. Sonnenburg J. L. Peterson D. A. Gordon J. I. (2005). Host-bacterial mutualism in the human intestine. *Science* 307 1915–1920. 10.1126/science.1104816 15790844

[B2] Baker R. Buckland A. Sheaves M. (2014). Fish gut content analysis: robust measures of diet composition. *Fish Fish.* 15 170–177.

[B3] Bakke I. Skjermo J. Vo T. A. Vadstein O. (2013). Live feed is not a major determinant of the microbiota associated with cod larvae (*Gadus morhua*). *Environ. Microbiol. Rep.* 5 537–548. 10.1111/1758-2229.12042 23864568

[B4] Bates A. E. McKelvie C. M. Sorte C. J. Morley S. A. Jones N. A. Mondon J. A. (2013). Geographical range, heat tolerance and invasion success in aquatic species. *Proc. Biol. Sci.* 280:20131958. 10.1098/rspb.2013.1958 24266040 PMC3813329

[B5] Bäumler A. J. Sperandio V. (2016). Interactions between the microbiota and pathogenic bacteria in the gut. *Nature* 535 85–93. 10.1038/nature18849 27383983 PMC5114849

[B6] Bolnick D. I. Snowberg L. K. Hirsch P. E. Lauber C. L. Org E. Parks B. (2014). Individual diet has sex-dependent effects on vertebrate gut microbiota. *Nat. Commun.* 5:4500. 10.1038/ncomms5500 25072318 PMC4279269

[B7] Butt R. L. Volkoff H. (2019). Gut Microbiota and energy homeostasis in fish. *Front. Endocrinol. (Lausanne)* 10:9. 10.3389/fendo.2019.00009 30733706 PMC6353785

[B8] Buttigieg P. L. Ramette A. J. F. M. E. (2014). A guide to statistical analysis in microbial ecology: a community-focused, living review of multivariate data analyses. *FEMS Microbiol. Ecol.* 90 543–550. 10.1111/1574-6941.12437 25314312

[B9] Caporaso J. G. Kuczynski J. Stombaugh J. Bittinger K. Bushman F. D. Costello E. K. (2010). QIIME allows analysis of high-throughput community sequencing data. *Nat. Methods* 7 335–336. 10.1038/nmeth.f.303 20383131 PMC3156573

[B10] Chen Y. Zhu X. Yang Y. Han D. Jin J. Xie S. (2014). Effect of dietary chitosan on growth performance, haematology, immune response, intestine morphology, intestine microbiota and disease resistance in gibel carp (*Carassius auratus* gibelio). *Aquaculture Nutrition* 20 532–546.

[B11] Clarke K. R. Warwick R. M. (1994). *Change in marine communities: An approach to statistical analysis and interpretation*. Plymouth: Plymouth marine laboratory, Natural environment research council.

[B12] Dai M. Wang L. Guo X. Zhai W. Li Q. He B. (2008). Nitrification and inorganic nitrogen distribution in a large perturbed river/estuarine system: the Pearl River Estuary. China. *Biogeosciences* 5 1227–1244.

[B13] David L. A. Maurice C. F. Carmody R. N. Gootenberg D. B. Button J. E. Wolfe B. E. (2014). Diet rapidly and reproducibly alters the human gut microbiome. *Nature* 505 559–563. 10.1038/nature12820 24336217 PMC3957428

[B14] Dickson R. P. Erb-Downward J. R. Falkowski N. R. Hunter E. M. Ashley S. L. Huffnagle G. B. (2018). The lung microbiota of healthy mice are highly variable, cluster by environment, and reflect variation in baseline lung innate immunity. *Am. J. Respir. Crit. Care Med.* 198 497–508. 10.1164/rccm.201711-2180OC 29533677 PMC6118022

[B15] Dixon P. (2003). VEGAN, a package of R functions for community ecology. *J. Vegetation Sci.* 14 927–930.

[B16] Donaldson G. P. Lee S. M. Mazmanian S. K. (2016). Gut biogeography of the bacterial microbiota. *Nat. Rev. Microbiol.* 14 20–32. 10.1038/nrmicro3552 26499895 PMC4837114

[B17] Edgar R. C. Haas B. J. Clemente J. C. Quince C. Knight R. (2011). UCHIME improves sensitivity and speed of chimera detection. *Bioinformatics* 27 2194–2200. 10.1093/bioinformatics/btr381 21700674 PMC3150044

[B18] Egerton S. Culloty S. Whooley J. Stanton C. Ross R. P. (2018). The gut microbiota of marine fish. *Front. Microbiol.* 9:873. 10.3389/fmicb.2018.00873 29780377 PMC5946678

[B19] Eichmiller J. J. Hamilton M. J. Staley C. Sadowsky M. J. Sorensen P. W. (2016). Environment shapes the fecal microbiome of invasive carp species. *Microbiome* 4:44. 10.1186/s40168-016-0190-191 PMC4981970 27514729

[B20] Fallani M. Amarri S. Uusijarvi A. Adam R. Khanna S. Aguilera M. (2011). Determinants of the human infant intestinal microbiota after the introduction of first complementary foods in infant samples from five European centres. *Microbiology (Reading)* 157(Pt 5), 1385–1392. 10.1099/mic.0.042143-42140 21330436

[B21] Fetissov S. O. (2017). Role of the gut microbiota in host appetite control: bacterial growth to animal feeding behaviour. *Nat. Rev. Endocrinol.* 13 11–25. 10.1038/nrendo.2016.150 27616451

[B22] Fujian Provincial Department of Ocean and Fisheries (2013). *Fujian province fishery statistical yearbook 2013*. Beijing: China Agriculture Press.

[B23] Flint H. J. Scott K. P. Duncan S. H. Louis P. Forano E. (2012). Microbial degradation of complex carbohydrates in the gut. *Gut Microbes* 3 289–306. 10.4161/gmic.19897 22572875 PMC3463488

[B24] Gang Y. Jian S. Q. Cao H. Wen C. Hu B. Mo P. (2018). Changes in microbiota along the intestine of grass carp (*Ctenopharyngodon idella*): community, interspecific interactions, and functions. *Aquaculture* 498 151–161.

[B25] Gatesoupe F. J. Huelvan C. Le Bayon N. Le Delliou H. Madec L. Mouchel O. (2016). The highly variable microbiota associated to intestinal mucosa correlates with growth and hypoxia resistance of sea bass, *Dicentrarchus labrax*, submitted to different nutritional histories. *BMC Microbiol.* 16:266. 10.1186/s12866-016-0885-2 27821062 PMC5100225

[B26] Ghoul M. Mitri S. (2016). The ecology and evolution of microbial competition. *Trends Microbiol.* 24 833–845. 10.1016/j.tim.2016.06.011 27546832

[B27] Gomez A. Petrzelkova K. J. Burns M. B. Yeoman C. J. Amato K. R. Vlckova K. (2016). Gut microbiome of coexisting BaAka pygmies and bantu reflects gradients of traditional subsistence patterns. *Cell Rep.* 14 2142–2153. 10.1016/j.celrep.2016.02.013 26923597

[B28] Günther C. Josenhans C. Wehkamp J. (2016). Crosstalk between microbiota, pathogens and the innate immune responses. *Int. J. Med. Microbiol.* 306 257–265. 10.1016/j.ijmm.2016.03.003 26996809

[B29] Han A. Dai M. Kao S. J. Gan J. Li Q. Wang L. (2012). Nutrient dynamics and biological consumption in a large continental shelf system under the influence of both a river plume and coastal upwelling. *Limnol. Oceanography* 57 486–502.

[B30] Hu Y. Sanders J. G. Łukasik P. D’Amelio C. L. Millar J. S. Vann D. R. (2018). Herbivorous turtle ants obtain essential nutrients from a conserved nitrogen-recycling gut microbiome. *Nat. Commun.* 9:964. 10.1038/s41467-018-03357-y 29511180 PMC5840417

[B31] Jeraldo P. Sipos M. Chia N. Brulc J. M. Dhillon A. S. Konkel M. E. (2012). Quantification of the relative roles of niche and neutral processes in structuring gastrointestinal microbiomes. *Proc. Natl. Acad. Sci. U S A.* 109 9692–9698. 10.1073/pnas.1206721109 22615407 PMC3382495

[B32] JGOFS, (1994). Protocols for the Joint Global Ocean Flux Study Core Measurements International JGOFS Report Series (Bergen: JGOFS International Project Office).

[B33] Jiang Y. Li S. Li R. Zhang J. Liu Y. Lv L. (2017a). Plant cultivars imprint the rhizosphere bacterial community composition and association networks. *Soil Biol. Biochem.* 109 145–155.

[B34] Jiang Y. Liu M. Zhang J. Chen Y. Chen X. Chen L. (2017b). Nematode grazing promotes bacterial community dynamics in soil at the aggregate level. *ISME J.* 11 2705–2717. 10.1038/ismej.2017.120 28742069 PMC5702727

[B35] Jiao S. Chen W. Wei G. (2017). Biogeography and ecological diversity patterns of rare and abundant bacteria in oil-contaminated soils. *Mol. Ecol.* 26 5305–5317. 10.1111/mec.14218 28665016

[B36] Jones J. DiBattista J. D. Stat M. Bunce M. Boyce M. C. Fairclough D. V. (2018). The microbiome of the gastrointestinal tract of a range-shifting marine herbivorous fish. *Front. Microbiol.* 9:2000. 10.3389/fmicb.2018.02000 30210475 PMC6121097

[B37] Kellogg C. A. Goldsmith D. B. Gray M. A. (2017). Biogeographic comparison of lophelia-associated bacterial communities in the western atlantic reveals conserved core microbiome. *Front. Microbiol.* 8:796. 10.3389/fmicb.2017.00796 28522997 PMC5415624

[B38] Kokou F. Sasson G. Friedman J. Eyal S. Ovadia O. Harpaz S. (2019). Core gut microbial communities are maintained by beneficial interactions and strain variability in fish. *Nat. Microbiol.* 4 2456–2465. 10.1038/s41564-019-0560-560 31548685

[B39] Koropatkin N. M. Cameron E. A. Martens E. C. (2012). How glycan metabolism shapes the human gut microbiota. *Nat. Rev. Microbiol.* 10 323–335. 10.1038/nrmicro2746 22491358 PMC4005082

[B40] Krause A. E. Frank K. A. Mason D. M. Ulanowicz R. E. Taylor W. W. (2003). Compartments revealed in food-web structure. *Nature* 426 282–285. 10.1038/nature02115 14628050

[B41] Langille M. G. I. Zaneveld J. Caporaso J. G. Mcdonald D. Knights D. Reyes J. (2013). Predictive functional profiling of microbial communities using 16S rRNA marker gene sequences. *Nat. Biotechnol.* 31 814–821. 23975157 10.1038/nbt.2676 PMC3819121

[B42] Layeghifard M. Hwang D. M. Guttman D. S. (2017). Disentangling interactions in the microbiome: a network perspective. *Trends Microbiol.* 25 217–228. 10.1016/j.tim.2016.11.008 27916383 PMC7172547

[B43] Le Doujet T. De Santi C. Klemetsen T. Hjerde E. Willassen N. P. Haugen P. (2019). Closely-related Photobacterium strains comprise the majority of bacteria in the gut of migrating Atlantic cod (*Gadus morhua*). *Microbiome* 7:64. 10.1186/s40168-019-0681-y 30995938 PMC6471968

[B44] Le Roy C. I. Beaumont M. Jackson M. A. Steves C. J. Spector T. D. Bell J. T. (2018). Heritable components of the human fecal microbiome are associated with visceral fat. *Gut Microbes* 9 61–67. 10.1080/19490976.2017.1356556 28767316 PMC5914912

[B45] Lek E. Fairclough D. V. Hall N. G. Hesp S. A. Potter I. C. (2012). Do the maximum sizes, ages and patterns of growth of three reef-dwelling labrid species at two latitudes differ in a manner conforming to the metabolic theory of ecology? *J. Fish Biol.* 81 1936–1962. 10.1111/j.1095-8649.2012.03446.x 23130692

[B46] Li X. Yu Y. Feng W. Yan Q. Gong Y. (2012). Host species as a strong determinant of the intestinal microbiota of fish larvae. *J. Microbiol.* 50 29–37. 10.1007/s12275-012-1340-1341 22367934

[B47] Liu X. Li Y. Wu Y. Huang B. Dai M. Fu F. (2017). Effects of elevated CO(2) on phytoplankton during a mesocosm experiment in the southern eutrophicated coastal water of China. *Sci. Rep.* 7:6868. 10.1038/s41598-017-07195-7198 PMC5537254 28761136

[B48] López Nadal A. Ikeda-Ohtsubo W. Sipkema D. Peggs D. McGurk C. Forlenza M. (2020). Feed, microbiota, and gut immunity: using the zebrafish model to understand fish health. *Front. Immunol.* 11:114. 10.3389/fimmu.2020.00114 32117265 PMC7014991

[B49] Lynch A. J. Cooke S. J. Deines A. M. Bower S. D. Bunnell D. B. Cowx I. G. (2016). The social, economic, and environmental importance of inland fish and fisheries. *Environ. Rev.* 24 115–121. 10.1139/er-2015-2064

[B50] Marchesi J. R. Adams D. H. Fava F. Hermes G. D. Hirschfield G. M. Hold G. (2016). The gut microbiota and host health: a new clinical frontier. *Gut* 65 330–339. 10.1136/gutjnl-2015-309990 26338727 PMC4752653

[B51] Martiny J. B. Eisen J. A. Penn K. Allison S. D. Horner-Devine M. C. (2011). Drivers of bacterial beta-diversity depend on spatial scale. *Proc. Natl. Acad. Sci. U S A.* 108 7850–7854. 10.1073/pnas.1016308108 21518859 PMC3093525

[B52] Mayer E. A. Tillisch K. Gupta A. (2015). Gut/brain axis and the microbiota. *J. Clin. Invest.* 125 926–938. 10.1172/jci76304 25689247 PMC4362231

[B53] McDonald D. Price M. N. Goodrich J. Nawrocki E. P. DeSantis T. Z. Probst A. (2012). An improved Greengenes taxonomy with explicit ranks for ecological and evolutionary analyses of bacteria and archaea. *ISME J.* 6 610–618. 10.1038/ismej.2011.139 22134646 PMC3280142

[B54] McDonald R. Zhang F. Watts J. E. Schreier H. J. (2015). Nitrogenase diversity and activity in the gastrointestinal tract of the wood-eating catfish Panaque nigrolineatus. *ISME J.* 9 2712–2724. 10.1038/ismej.2015.65 25909976 PMC4817639

[B55] McElhanon B. O. McCracken C. Karpen S. Sharp W. G. (2014). Gastrointestinal symptoms in autism spectrum disorder: a meta-analysis. *Pediatrics* 133 872–883. 10.1542/peds.2013-3995 24777214

[B56] Michael P. J. Hyndes G. A. Vanderklift M. A. Verges A. (2013). Identity and behaviour of herbivorous fish influence large-scale spatial patterns of macroalgal herbivory in a coral reef. *Mar. Ecol. Progress Series* 482 227–240.

[B57] Ministry of Agriculture and Rural Affairs (2019). *China Fishery Statistical Yearbook 2019.* China: China Agriculture Press.

[B58] Na X. Xia X. Liu T. Hu L. Zhu B. Zhang X. (2014). Characteristics of bacterial community in the water and surface sediment of the Yellow River, China, the largest turbid river in the world. *J. Soils Sediments* 14 1894–1904.

[B59] Navarrete P. Magne F. Araneda C. Fuentes P. Barros L. Opazo R. (2012). PCR-TTGE analysis of 16S rRNA from rainbow trout (Oncorhynchus mykiss) gut microbiota reveals host-specific communities of active bacteria. *PLoS One* 7:e31335. 10.1371/journal.pone.0031335 22393360 PMC3290605

[B60] Nielsen S. Wilkes Walburn J. Vergés A. Thomas T. Egan S. (2017). Microbiome patterns across the gastrointestinal tract of the rabbitfish Siganus fuscescens. *PeerJ* 5:e3317. 10.7717/peerj.3317 28533966 PMC5437856

[B61] Otles S. (2014). *Aquaculture Nutrition: Gut Health, Probiotics and Prebiotics.* Boston, MA: Houghton Mifflin Riverside Press, 146–167.

[B62] Pai S. C. Tsau Y. J. Yang T. I. J. A. C. A. (2001). pH and buffering capacity problems involved in the determination of ammonia in saline water using the indophenol blue spectrophotometric method. 434 209–216.

[B63] Parks D. H. Tyson G. W. Hugenholtz P. Beiko R. G. (2014). STAMP: statistical analysis of taxonomic and functional profiles. *Bioinformatics* 30 3123–3124. 10.1093/bioinformatics/btu494 25061070 PMC4609014

[B64] Pérez T. Balcázar J. L. Ruiz-Zarzuela I. Halaihel N. Vendrell D. de Blas I. (2010). Host-microbiota interactions within the fish intestinal ecosystem. *Mucosal Immunol.* 3 355–360. 10.1038/mi.2010.12 20237466

[B65] Pitt J. M. (1997). *The feeding Ecology of Rabbitfish (Siganidae) at Green Island Reef: Ontogenetic and Interspecific Differences in Diet, Morphology and Habitat Utilisation.* PhD thesis, Douglas QLD: James Cook University of North Queensland.

[B66] Porra R. J. (2002). The chequered history of the development and use of simultaneous equations for the accurate determination of chlorophylls a and b. *Photosynthesis Res.* 73 149–156. 10.1023/A:1020470224740 16245116

[B67] Propheter D. C. Chara A. L. Harris T. A. Ruhn K. A. Hooper L. V. (2017). Resistin-like molecule β is a bactericidal protein that promotes spatial segregation of the microbiota and the colonic epithelium. *Proc. Natl. Acad. Sci. U S A.* 114 11027–11033. 10.1073/pnas.1711395114 28973871 PMC5651776

[B68] Rehman A. Rausch P. Wang J. Skieceviciene J. Kiudelis G. Bhagalia K. (2016). Geographical patterns of the standing and active human gut microbiome in health and IBD. *Gut* 65 238–248. 10.1136/gutjnl-2014-308341 25567118

[B69] Rinninella E. Raoul P. Cintoni M. Franceschi F. Miggiano G. A. D. Gasbarrini A. (2019). What is the healthy gut microbiota composition? a changing ecosystem across age, environment, diet, and diseases. *Microorganisms* 7:14. 10.3390/microorganisms7010014 30634578 PMC6351938

[B70] Schmidt E. Mykytczuk N. Schulte-Hostedde A. I. (2019). Effects of the captive and wild environment on diversity of the gut microbiome of deer mice (*Peromyscus maniculatus*). *ISME J.* 13 1293–1305. 10.1038/s41396-019-0345-348 30664674 PMC6474230

[B71] Schoeler M. Caesar R. (2019). Dietary lipids, gut microbiota and lipid metabolism. *Rev. Endocr. Metab. Disord.* 20 461–472. 10.1007/s11154-019-09512-9510 31707624 PMC6938793

[B72] Schulfer A. F. Battaglia T. Alvarez Y. Bijnens L. Ruiz V. E. Ho M. (2018). Intergenerational transfer of antibiotic-perturbed microbiota enhances colitis in susceptible mice. *Nat. Microbiol.* 3 234–242. 10.1038/s41564-017-0075-75 29180726 PMC5780248

[B73] Segata N. Izard J. Waldron L. Gevers D. Miropolsky L. Garrett W. S. (2011). Metagenomic biomarker discovery and explanation. *Genome Biol.* 12:R60. 10.1186/gb-2011-12-6-r60 21702898 PMC3218848

[B74] Shaani Y. Zehavi T. Eyal S. Miron J. Mizrahi I. (2018). Microbiome niche modification drives diurnal rumen community assembly, overpowering individual variability and diet effects. *ISME J.* 12 2446–2457. 10.1038/s41396-018-0203-200 29921849 PMC6154959

[B75] Shabat S. K. Sasson G. Doron-Faigenboim A. Durman T. Yaacoby S. Berg Miller M. E. (2016). Specific microbiome-dependent mechanisms underlie the energy harvest efficiency of ruminants. *ISME J.* 10 2958–2972. 10.1038/ismej.2016.62 27152936 PMC5148187

[B76] Sharon G. Sampson T. R. Geschwind D. H. Mazmanian S. K. (2016). The central nervous system and the gut microbiome. *Cell* 167 915–932. 10.1016/j.cell.2016.10.027 27814521 PMC5127403

[B77] Shenhav L. Thompson M. Joseph T. A. Briscoe L. Furman O. Bogumil D. (2019). FEAST: fast expectation-maximization for microbial source tracking. *Nat. Methods* 16 627–632. 10.1038/s41592-019-0431-x 31182859 PMC8535041

[B78] Smith C. C. Snowberg L. K. Gregory Caporaso J. Knight R. Bolnick D. I. (2015). Dietary input of microbes and host genetic variation shape among-population differences in stickleback gut microbiota. *ISME J.* 9 2515–2526. 10.1038/ismej.2015.64 25909977 PMC4611514

[B79] Sonnenburg J. L. Bäckhed F. (2016). Diet-microbiota interactions as moderators of human metabolism. *Nature* 535 56–64. 10.1038/nature18846 27383980 PMC5991619

[B80] Sullam K. E. Essinger S. D. Lozupone C. A. O’Connor M. P. Rosen G. L. Knight R. (2012). Environmental and ecological factors that shape the gut bacterial communities of fish: a meta-analysis. *Mol. Ecol.* 21 3363–3378. 10.1111/j.1365-294X.2012.05552.x 22486918 PMC3882143

[B81] Tlaskalová-Hogenová H. Stěpánková R. Kozáková H. Hudcovic T. Vannucci L. Tučková L. (2011). The role of gut microbiota (commensal bacteria) and the mucosal barrier in the pathogenesis of inflammatory and autoimmune diseases and cancer: contribution of germ-free and gnotobiotic animal models of human diseases. *Cell Mol. Immunol.* 8 110–120. 10.1038/cmi.2010.67 21278760 PMC4003137

[B82] Tuomisto H. Ruokolainen K. Yli-Halla M. (2003). Dispersal, environment, and floristic variation of western Amazonian forests. *Science* 299 241–244. 10.1126/science.1078037 12522248

[B83] Turnbaugh P. J. Hamady M. Yatsunenko T. Cantarel B. L. Duncan A. Ley R. E. (2009). A core gut microbiome in obese and lean twins. *Nature* 457 480–484. 10.1038/nature07540 19043404 PMC2677729

[B84] Wang J. Jiang Y. Li X. Tao H. Yang Y. Hu S. (2016a). Dietary protein requirement of juvenile red spotted grouper (*Epinephelus akaara*). *Aquaculture* 450 289–294.

[B85] Wang J. Thingholm L. B. Skiecevičienė J. Rausch P. Kummen M. Hov J. R. (2016b). Genome-wide association analysis identifies variation in vitamin D receptor and other host factors influencing the gut microbiota. *Nat. Genet.* 48 1396–1406. 10.1038/ng.3695 27723756 PMC5626933

[B86] Wen C. Yan W. Sun C. Ji C. Zhou Q. Zhang D. (2019). The gut microbiota is largely independent of host genetics in regulating fat deposition in chickens. *ISME J.* 13 1422–1436. 10.1038/s41396-019-0367-362 30728470 PMC6775986

[B87] Xia J. H. Lin G. Fu G. Wan Z. Y. Lee M. M. Wang L. (2014). The intestinal microbiome of fish under starvation. *BMC Genomics* 15:266. 10.1186/1471-2164-15-266 24708260 PMC4234480

[B88] Xing M. Hou Z. Yuan J. Liu Y. Qu Y. Liu B. (2013). Taxonomic and functional metagenomic profiling of gastrointestinal tract microbiome of the farmed adult turbot (*Scophthalmus maximus*). *FEMS Microbiol. Ecol.* 86 432–443. 10.1111/1574-6941.12174 23802730

[B89] Yan Q. Li J. Yu Y. Wang J. He Z. Van Nostrand J. D. (2016). Environmental filtering decreases with fish development for the assembly of gut microbiota. *Environ. Microbiol.* 18 4739–4754. 10.1111/1462-2920.13365 27130138

[B90] Zeevi D. Korem T. Godneva A. Bar N. Kurilshikov A. Lotan-Pompan M. (2019). Structural variation in the gut microbiome associates with host health. *Nature* 568 43–48. 10.1038/s41586-019-1065-y 30918406

[B91] Zhao Z. Pan Y. Jiang J. Gao S. Sun H. Dong Y. (2018). Unrevealing variation of microbial communities and correlation with environmental variables in a full culture-cycle of *Undaria pinnatifida*. *Mar. Environ. Res.* 139 46–56. 10.1016/j.marenvres.2018.05.012 29754736

[B92] Zhou L. Liu L. Chen W. Y. Sun J. J. Hou S. W. Kuang T. X. (2020). Stochastic determination of the spatial variation of potentially pathogenic bacteria communities in a large subtropical river. *Environ. Pollut.* 264:114683. 10.1016/j.envpol.2020.114683 32388300

[B93] Zoetendal E. G. Raes J. van den Bogert B. Arumugam M. Booijink C. C. Troost F. J. (2012). The human small intestinal microbiota is driven by rapid uptake and conversion of simple carbohydrates. *ISME J* 6 1415–1426. 10.1038/ismej.2011.212 22258098 PMC3379644

