# Supplementary material for: Multi-dimensional investigation and distribution characteristics analysis of gut microbiota of different marine fish in Fujian Province of China
Source: Front Microbiol. 2022 Sep 27;13:918191. doi: 10.3389/fmicb.2022.918191 (PMC9551612; doi:10.3389/fmicb.2022.918191)
Supplement: Supplementary file 1 [file Data_Sheet_1.docx]

**Multi-dimensional investigation and distribution characteristics analysis of gut microbiota of different marine fish in Fujian Province of China**

**Supplementary Figures**

**
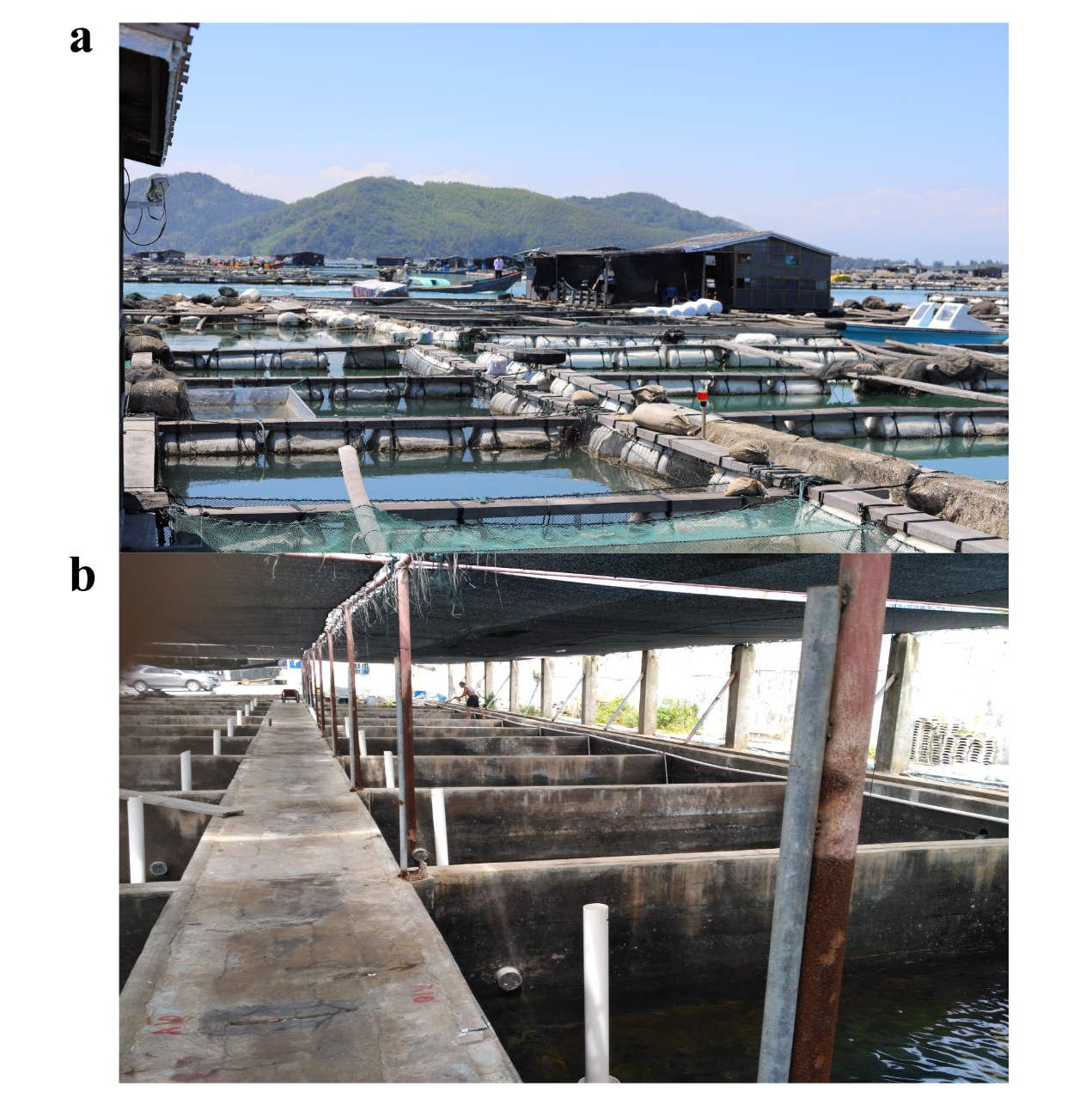
**

**Supplementary Figure 1.** The culture environment of different habitats. **a**, the culture environment of cage aquaculture company. **b**, the culture environment of cement pool aquaculture.


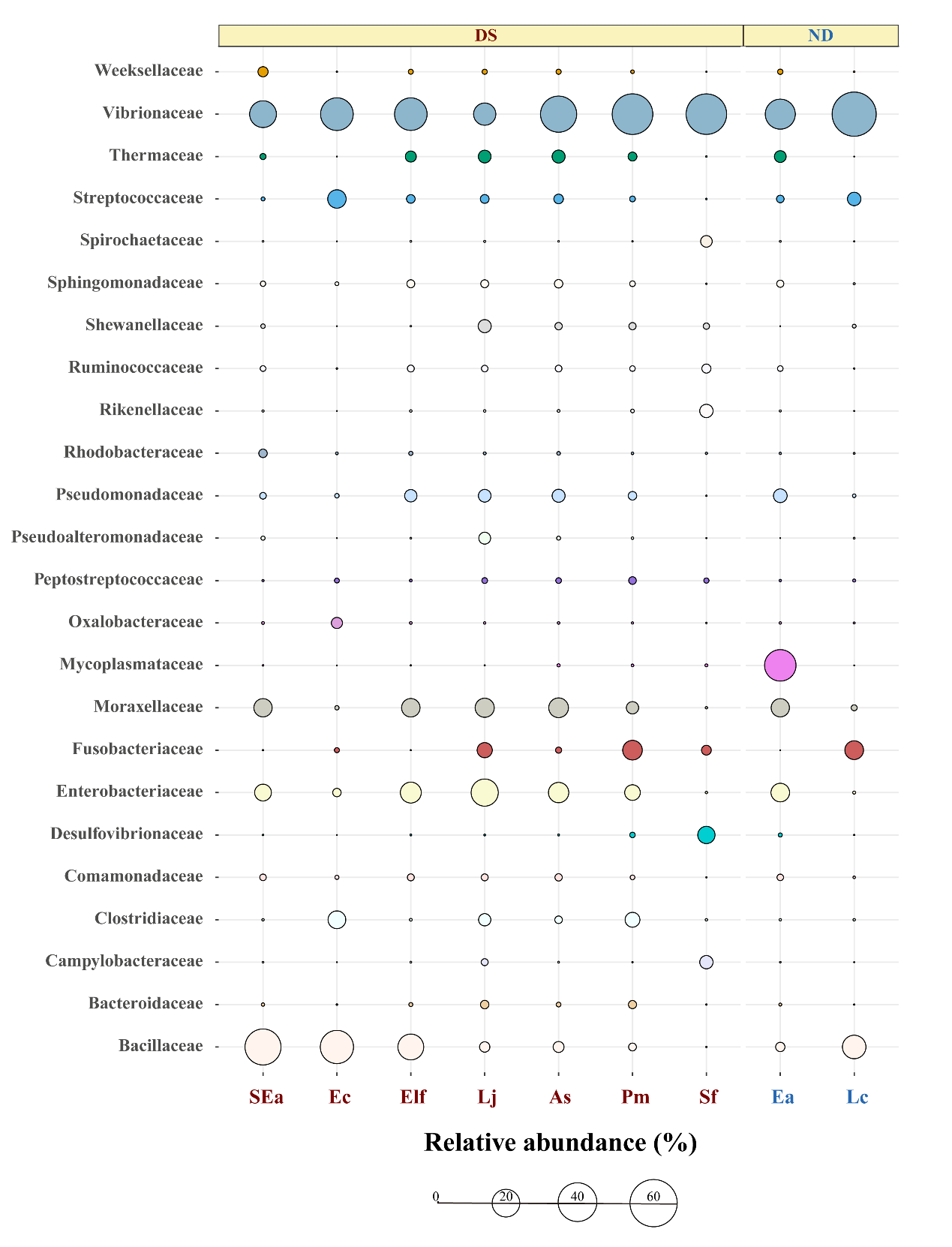


**Supplementary Figure 2**. Balloon plot showing the abundance of the core specificity communities across different fish species both in DS and ND at the family level. (each group represents 9 fish individuals mixed by 12 sample individuals).


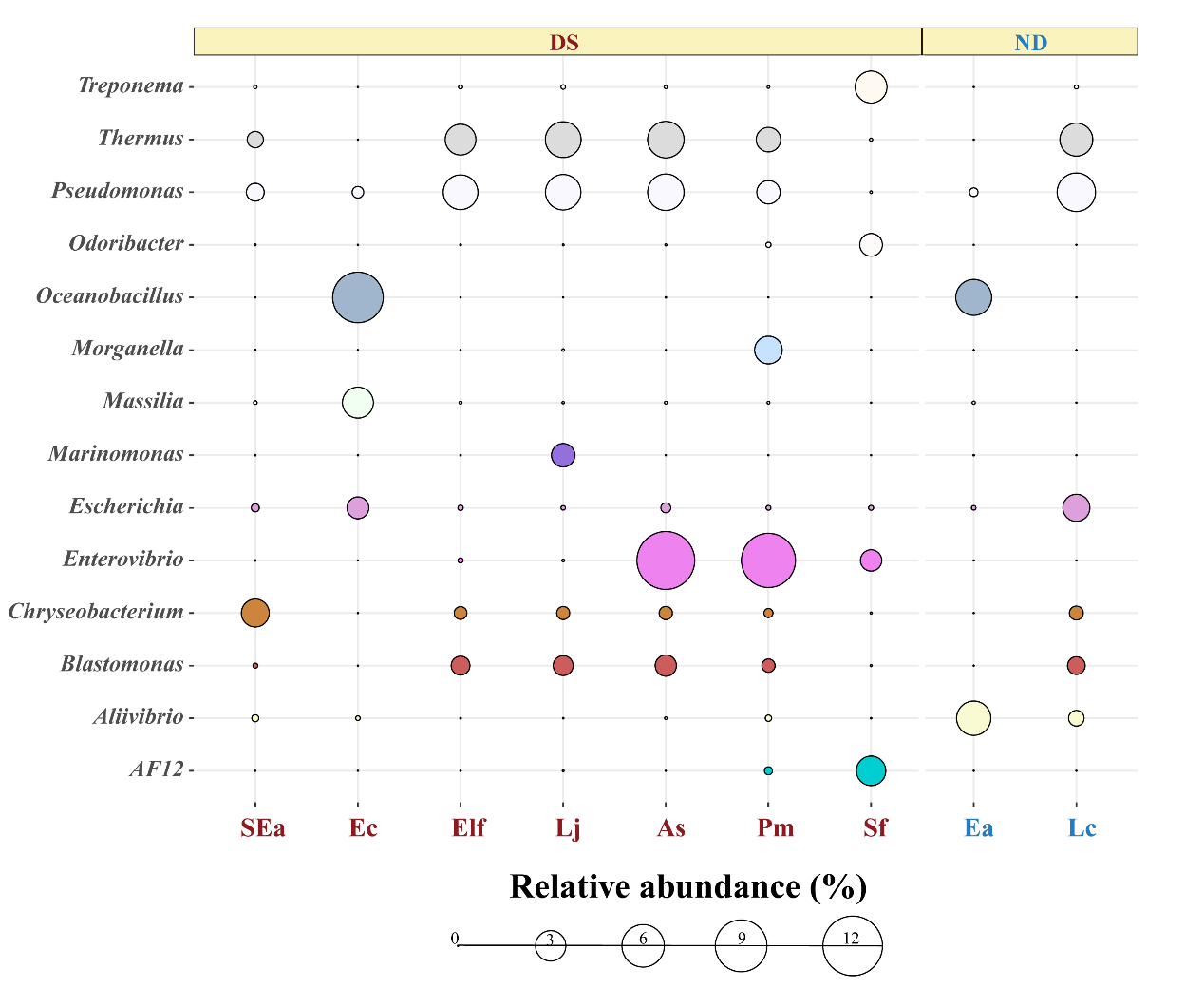


**Supplementary Figure 3**. Balloon plot showing the core specificity communities with low abundance across different fish species both in DS and ND at the genus level. (each group represents 9 fish individuals mixed by 12 sample individuals).


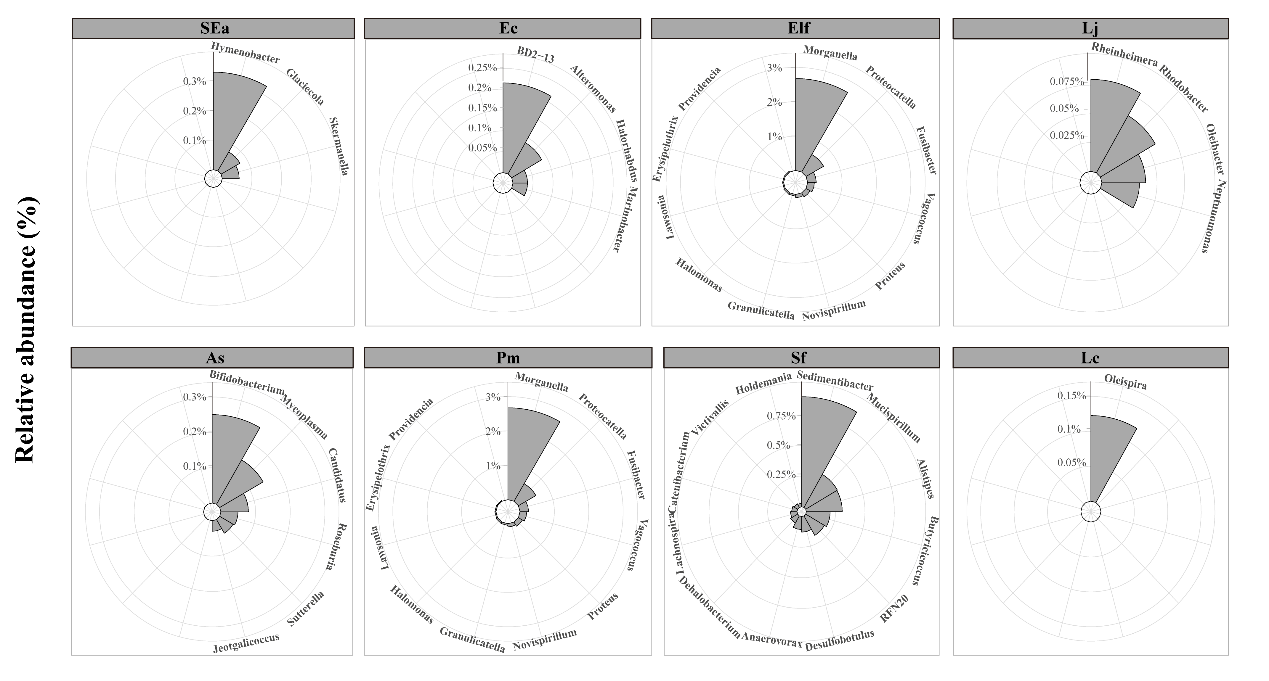


**Supplementary Figure 4**. Radar diagrams showing the relative abundance of unique microbial communities across different fish species at the genus level. (each group represents 9 fish individuals mixed by 12 sample individuals).


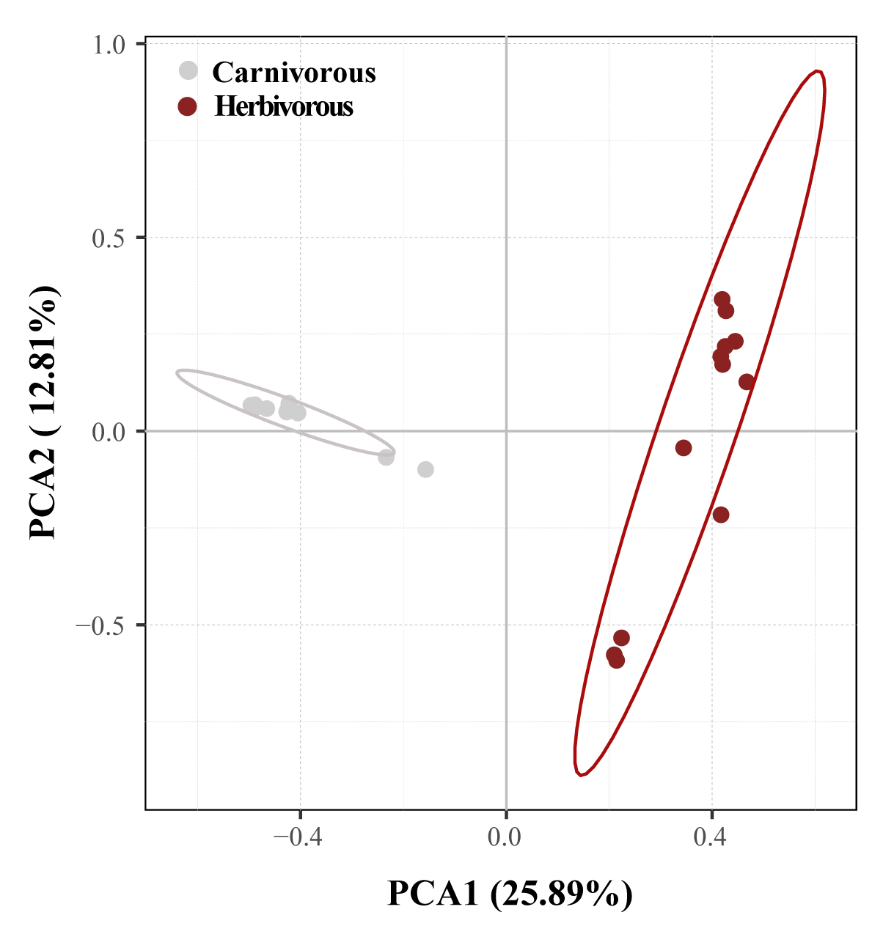


**Supplementary Figure 5**. Principal components analysis (PCA) plot generated using OTU metrics based on the Bray–Curtis dissimilarities showing the differences in bacterial community composition between herbivorous and carnivorous fish. Each point of herbivorous represents a sample. Each point of carnivorous represents a fish individual mixed by 12 sample individuals. Differences were assessed by PERMANOVA and significance was established at p < 0.05 (PERMANOVA, R-statistic = 0.4568541 P = 0.001, 95% confidence interval).

**Supplementary Figure 6**. The divergence of microbial communities between carnivorous and vegetarian fishes was shown as a combination of PCA plots and box plots. The PCA plots generated using OTU metrics based on the Bray–Curtis dissimilarities. Each point represents a sample. Differences were assessed by PERMANOVA and significance was established at p < 0.05. The box plots showing the relative abundance of Sf and other carnivorous fish groups, each group represents 9 fish individuals mixed by 12 sample individuals


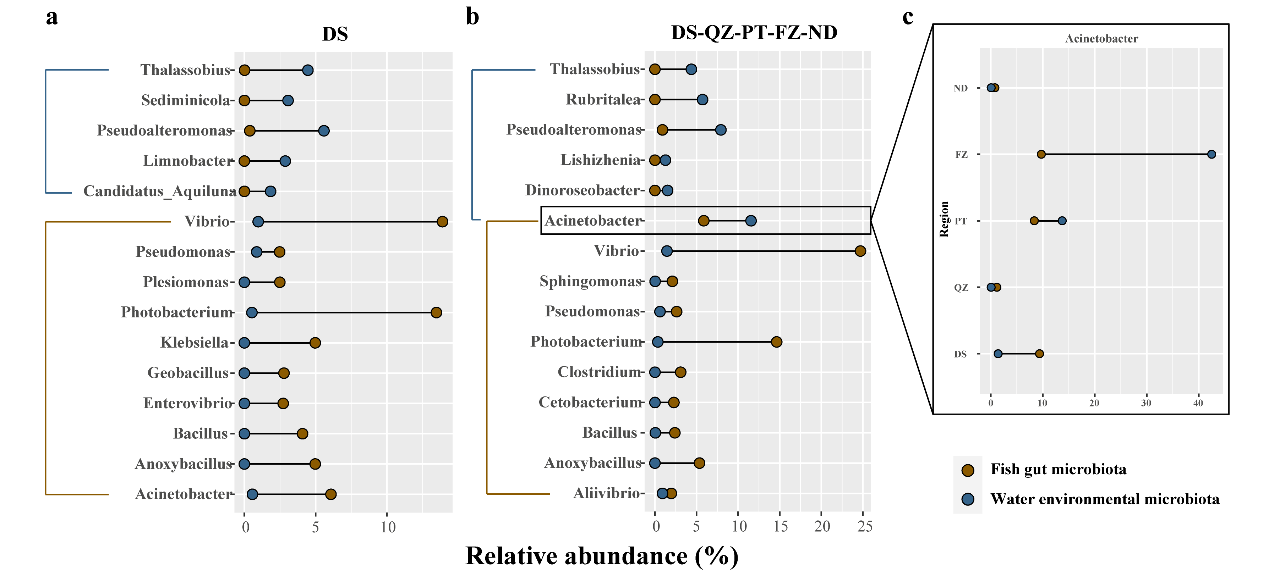


**Supplementary Figure 7**. The divergence of core microbial communities between fish gut and water environment was shown as dumbbell plots. The relative abundance of dominant microbial genus of water environment with top 5 and fish gut with top 10 were plotted. **a**, the divergence of core microbial communities between fish gut and water environment in DS region with seven fish species, all dominant microbial genus were significantly higher than the comparison group. **b**, the divergence of core microbial communities between fish gut and water environment in five regions (DS, QZ, PT, FZ and ND) with one fish species (Ea), the abundance of the top ten dominant microbial genus in fish gut were significantly higher than that in the water environment, and the abundance of the top five dominant microbial genus in the water environment were significantly higher than that in the fish gut, except *Acinetobacter.* **c**, the abundance of *Acinetobacter* was high in both fish gut and water environment in the PT and FZ regions.


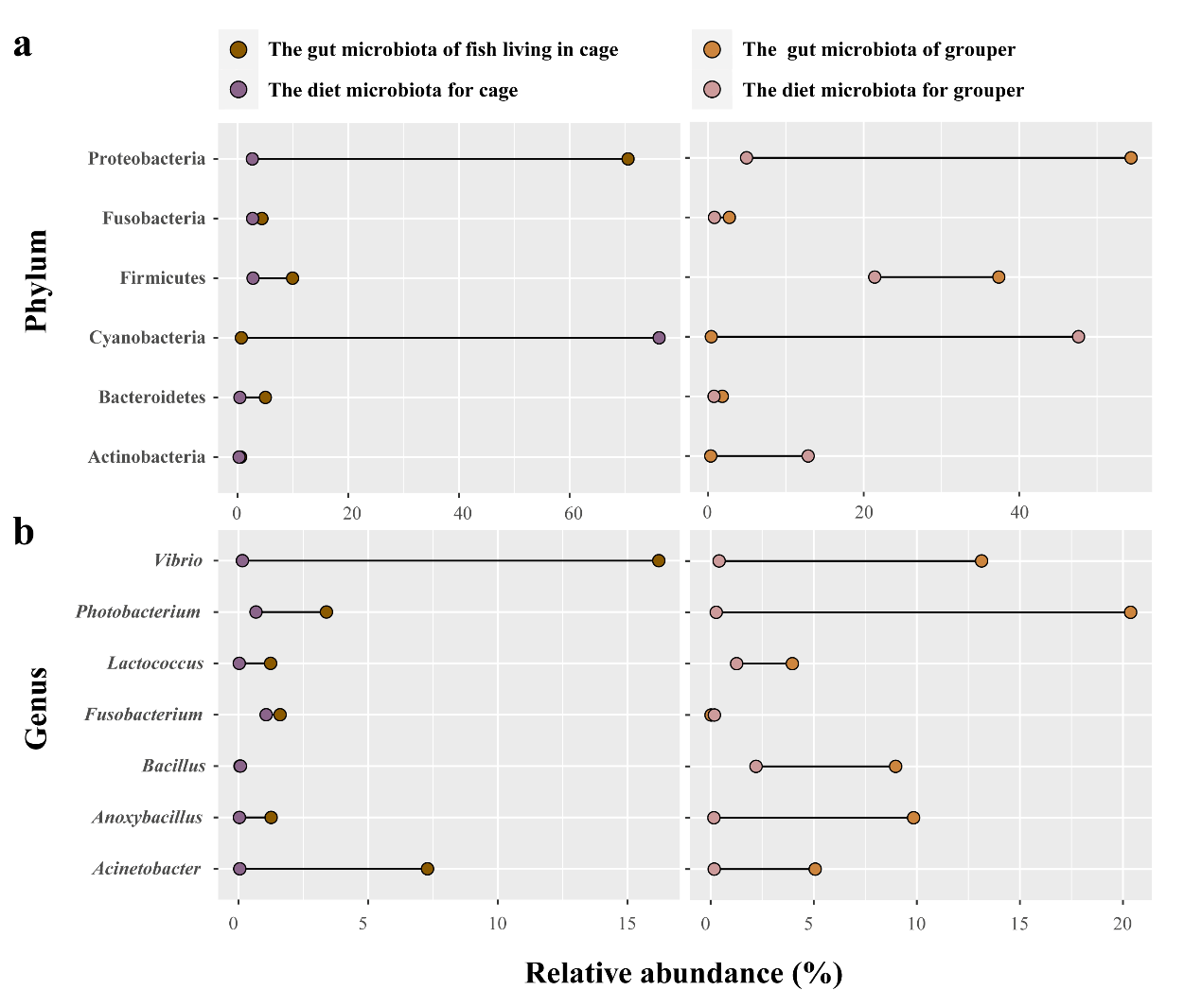


**Supplementary Figure 8**. The divergence of core microbial communities between fish gut and diet was shown as dumbbell plots. The relative abundance of dominant microbial phylum and genus of water environment belonging top 10 were plotted. **a**, the divergence of core microbial communities between fish gut and diet with two feeding methods at phylum level, although Firmicutes showed similar distribution trend to a certain extent in both feeding group, other core microbial showed significantly divergence between fish gut and diet, such as the most abundant Cyanobacteria in diet (47.6-76.1% in diet and 0.4-0.6% in fish gut) and the most abundant Proteobacteria in fish gut (2.6-4.9% in diet and 54.3-70.5% in fish gut). **b**, the divergence of core microbial communities between fish gut and diet with two feeding methods at phylum level, all dominant microbial genus showed significantly divergence between fish gut and diet.


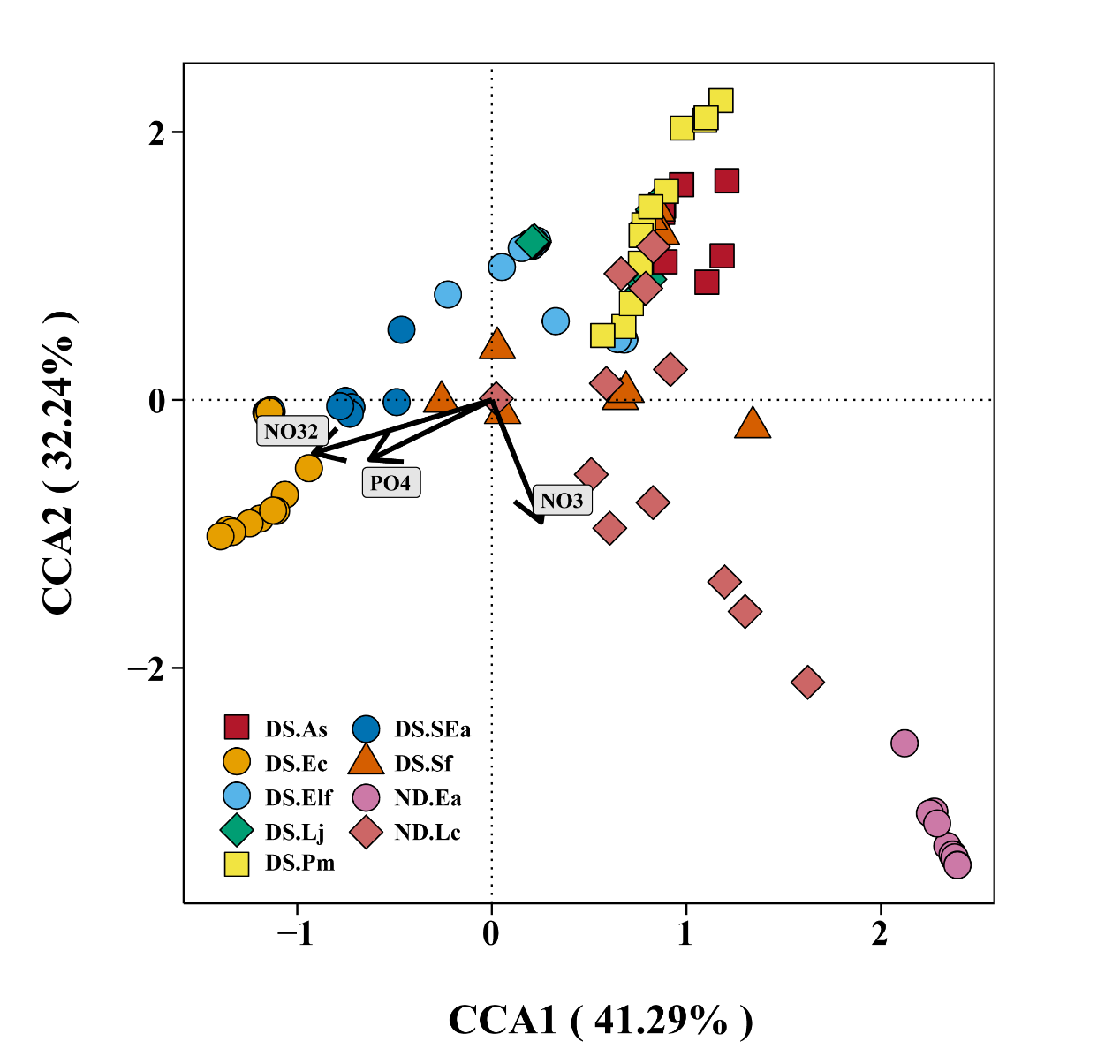


**Supplementary Figure 9**. The canonical correspondence analysis evaluated the relationship between the microbial communities of different fish species and environmental factors both in DS and ND. ANOSIM *R*=0.5652, *p*=0.001.


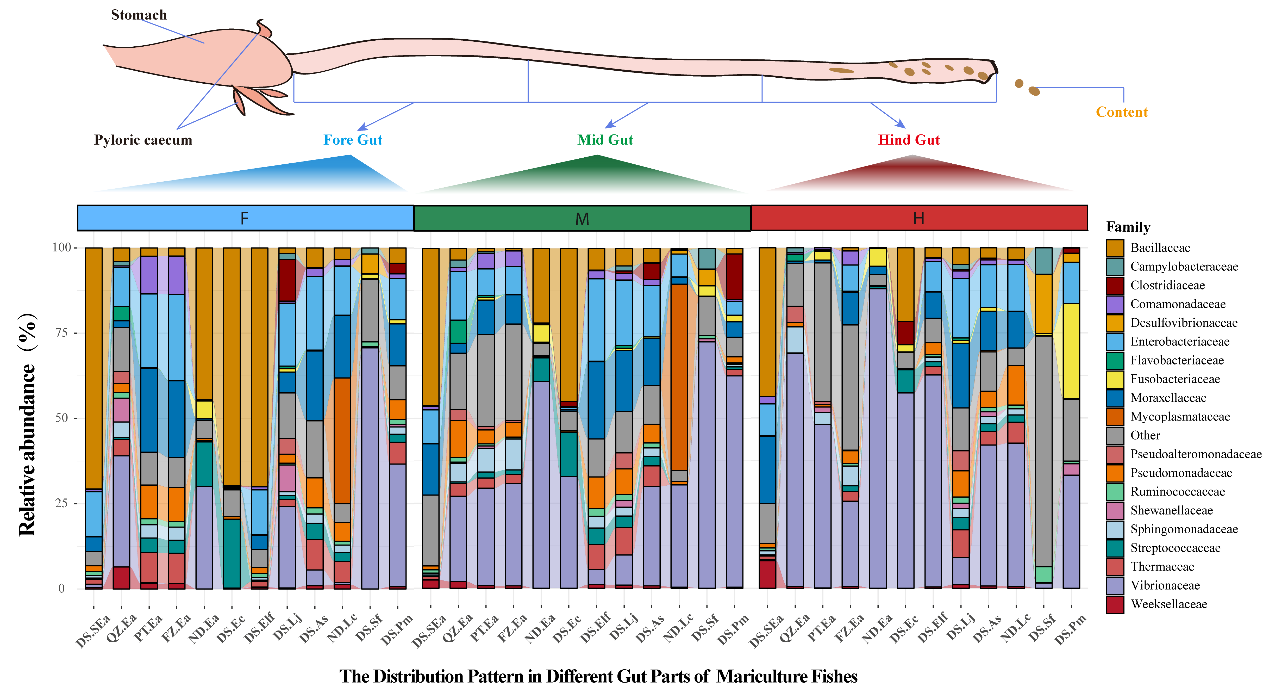


**Supplementary Figure 10**. The relative abundance of the microbial communities at the Family level found in different gut compartments (foregut, midgut and hindgut). Each group represents 9 fish individuals with a parallel sample mixed by three individuals. Only the dominant microbial family with top 20 of the sites are plotted.


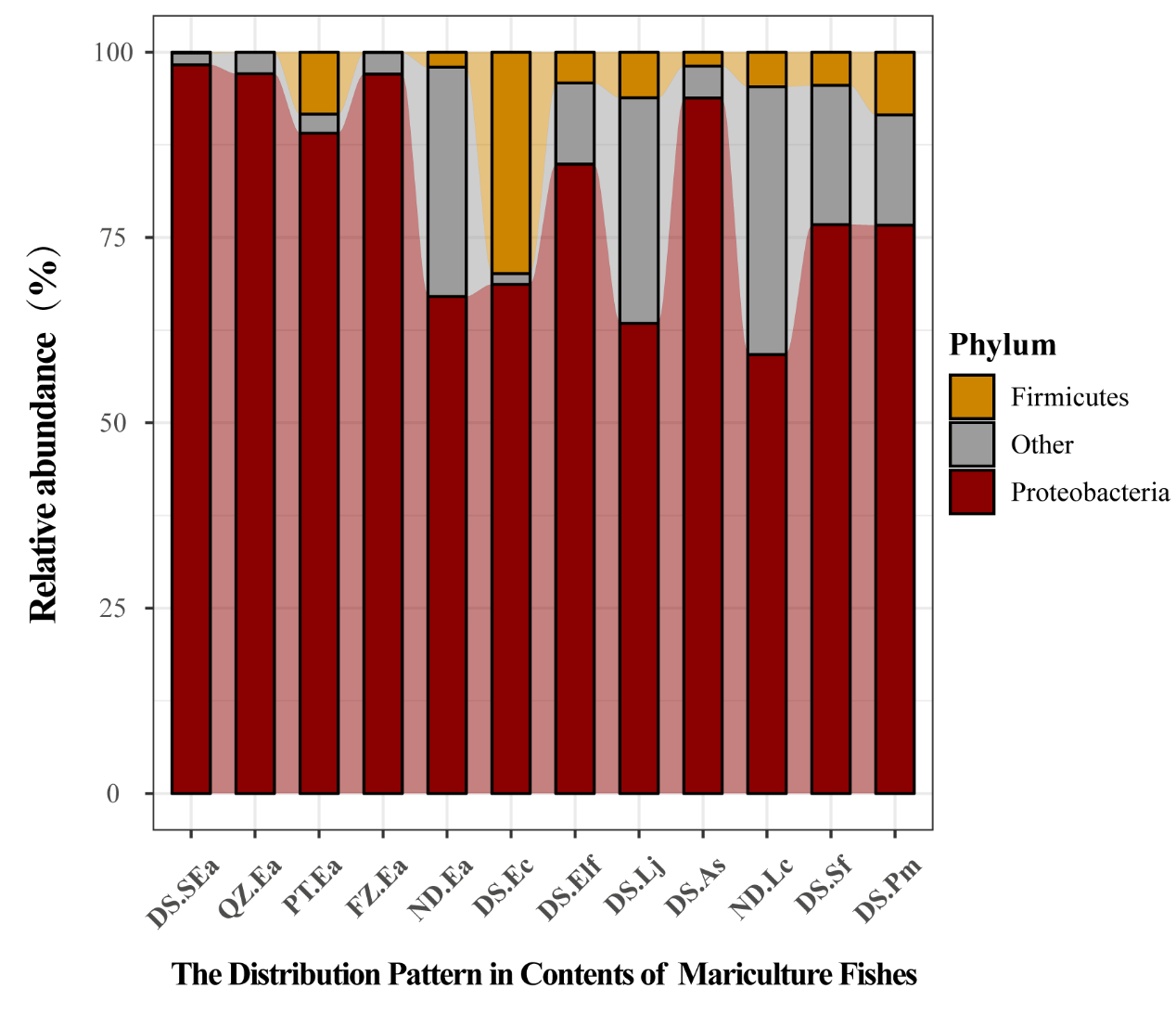


**Supplementary Figure 11**. The relative abundance of the microbial communities at the Phylum level found in content. Each group represents 9 fish individuals with a parallel sample mixed by three individuals. Only Proteobacteria and Firmicutes of contents are plotted to match the Fig4.


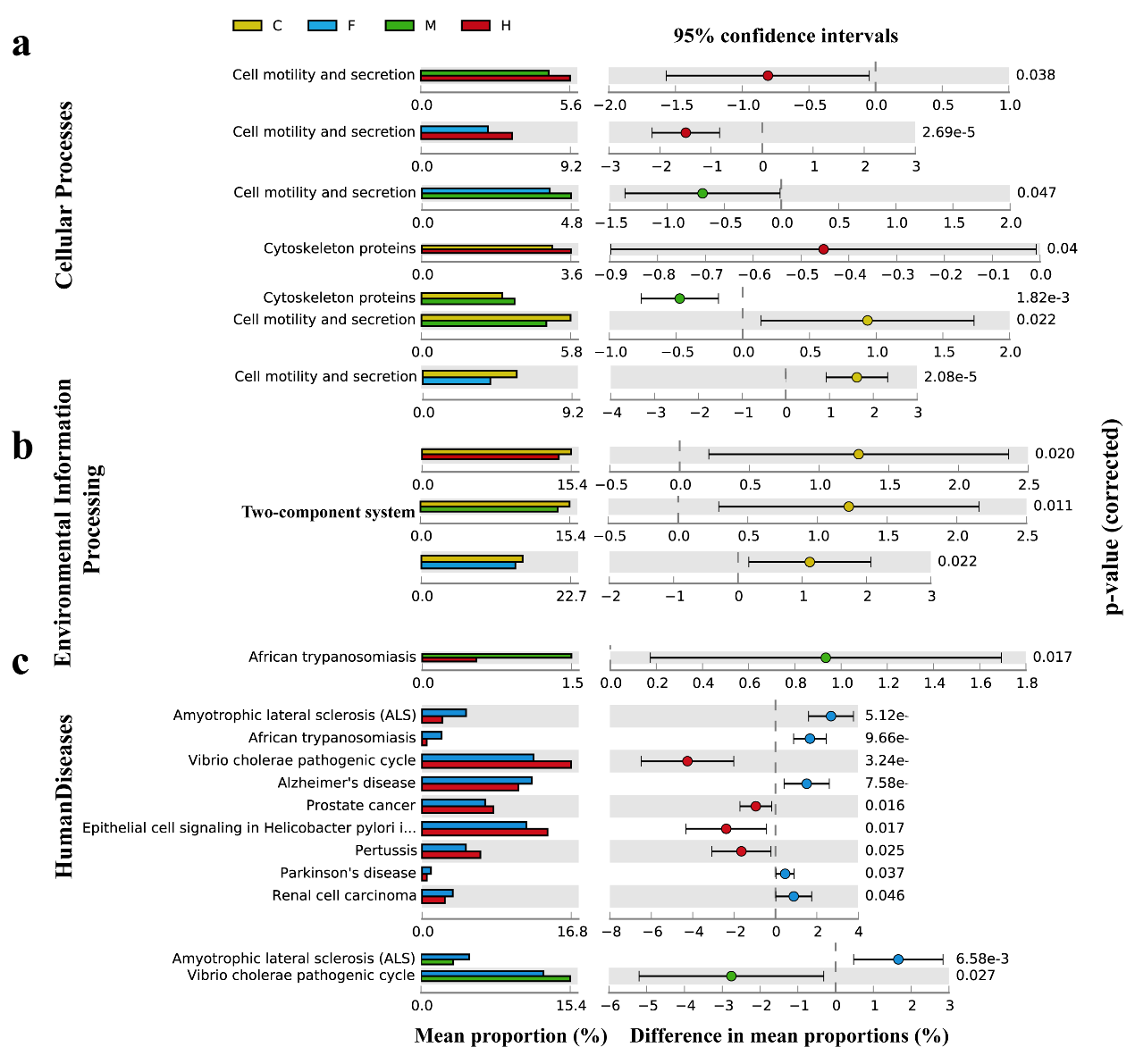


**Supplementary Figure 12a-c**. Differences in the abundance of KEGG functions at level 3 in samples among different gut parts (foregut, midgut, hindgut and content), p<0.05. **a**, the cellular processes related functions. **b**, the environmental information processing related functions. **c**, the human diseases related functions.


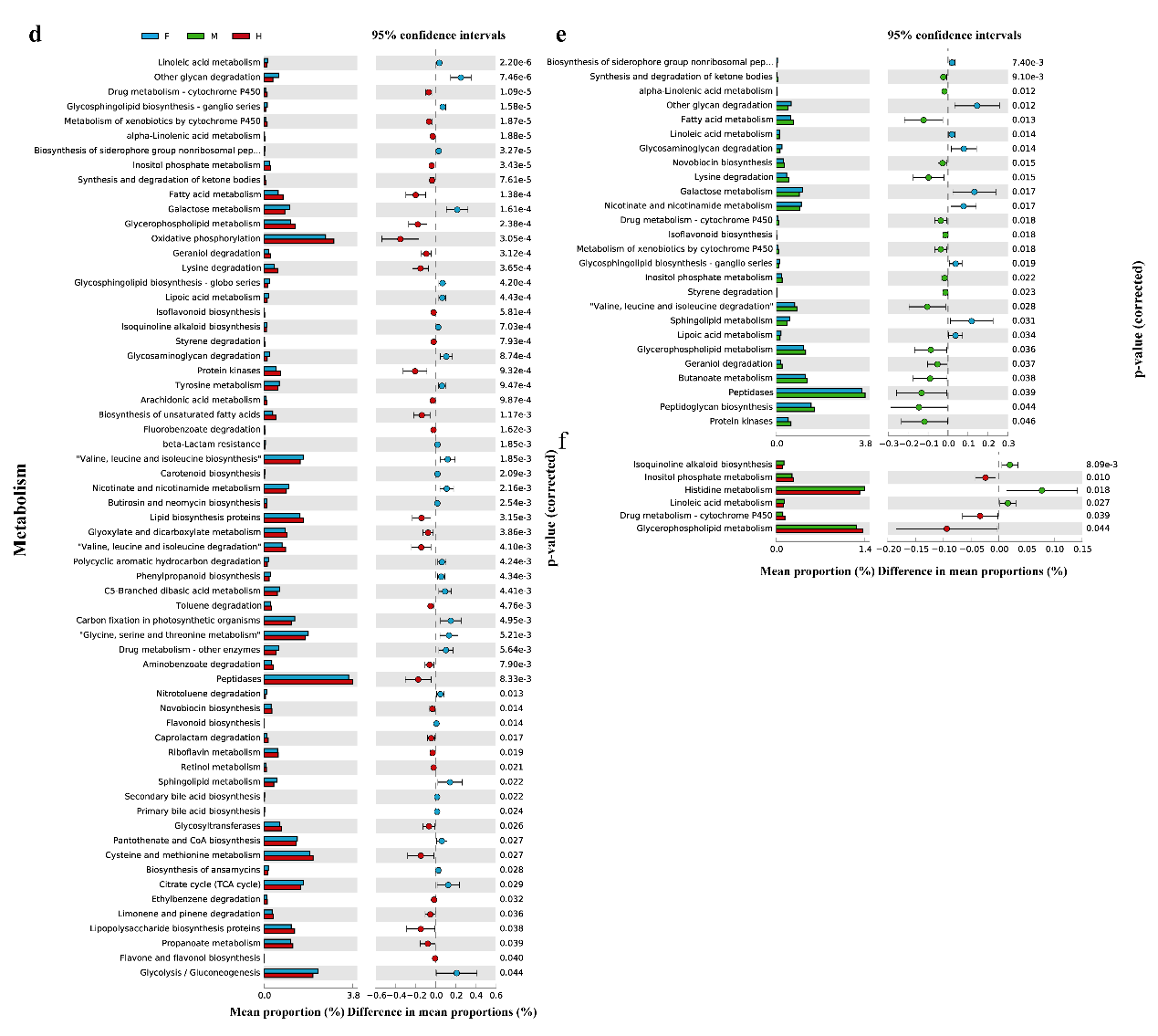


**Supplementary Figure 12d-f**. Differences in the abundance of KEGG functions at level 3 in samples among different gut parts (foregut, midgut, hindgut and content), p<0.05. **d**, the metabolism related functions between foregut and hindgut. **e**, the metabolism related functions between foregut and midgut. **f**, the metabolism related functions between midgut and hindgut.
